# Loss of microRNA-27b contributes to breast cancer stem cell generation by activating ENPP1

**DOI:** 10.1038/ncomms8318

**Published:** 2015-06-12

**Authors:** Ryou-u Takahashi, Hiroaki Miyazaki, Fumitaka Takeshita, Yusuke Yamamoto, Kaho Minoura, Makiko Ono, Makoto Kodaira, Kenji Tamura, Masaki Mori, Takahiro Ochiya

**Affiliations:** 1 Division of Molecular and Cellular Medicine, National Cancer Center Research Institute, 1-1, Tsukiji 5-chome, Tokyo 104-0045, Japan; 2 Genomic Application Group, Japan Diagnostics and Genomics, Agilent Technologies Japan Ltd., 9-1, Tokyo 192-8510, Japan; 3 Department of Breast and Medical Oncology, National Cancer Center Hospital, 1-1, Tsukiji 5-chome, Tokyo 104-0045, Japan; 4 Department of Gastroenterological Surgery, Graduated School of Medicine, Osaka University, Osaka 565-0871, Japan

## Abstract

Cancer stem cells (CSCs) have been identified in various types of cancer; however, the mechanisms by which cells acquire CSC properties such as drug resistance and tumour seeding ability are not fully understood. Here, we identified microRNA-27b (miR-27b) as a key regulator for the generation of a side-population in breast cancer cells that showed CSC properties, and also found that the anti-type II diabetes (T2D) drug metformin reduced this side-population via miR-27b-mediated repression of ectonucleotide pyrophosphatase/phosphodiesterase family member 1 (ENPP1), which is involved in T2D development. ENPP1 induced the generation of the side-population via upregulation of the ABCG2 transporter. ENPP1 was also identified as a substrate of the 26S proteasome, the activity of which is downregulated in CSCs. Overall, these results demonstrate that a T2D-associated gene plays an important role in tumour development and that its expression is strictly controlled at the mRNA and protein levels.

## Abstract (web summary)

MicroRNAs have a role in the acquisition of stem cell-like properties of cancer cells. Here the authors show that microRNA-27b mediates generation of a side-population of breast cancer stem cells, in part by regulating the protein ENPP1, which has been previously linked to the development of diabetes.

## Graphical abstract

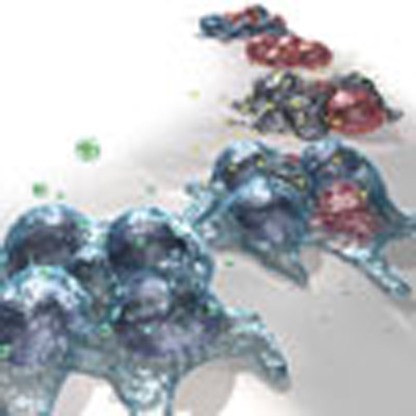

Accumulating evidence suggests that cancer and metabolic disease share common biological mechanisms^1^,^2^,^3^. Because tumours need to regulate their proliferation to cope with environmental challenges, such as hypoxia, nutrient starvation and anchorage-independent conditions, cancer cells have dramatically altered metabolic circuitry that is caused by oncogenic mutations selected during tumour initiation and development^4^,^5^. Several genes involved in type II diabetes (T2D) are associated with tumour initiation or cell cycle regulation^6^,^7^. In addition, microRNAs (miRNAs) are emerging as regulators of metabolic and malignant transformation during tumour development^8^,^9^. Recent studies demonstrated that miRNAs play important roles in the acquisition of cancer stem cell (CSC) properties such as tumour initiation, asymmetric cell division and drug resistance^10^,^11^.

CSCs, which are operationally defined as cells that form tumours after transplantation into immune-deficient mice, show self-renewal and drug tolerance^12^. In breast cancer, CD44^high^/CD24^low^ cells, aldehyde dehydrogenase (ALDH)-positive cells and side-population cells (known as the SP fraction) have been reported as CSCs or tumour-initiating cells^12^,^13^,^14^. The SP fraction has an enhanced ability to efflux small molecules, including anti-cancer agents, and this efflux activity is regulated by ABC transporters such as ABCB1 and ABCG2 (refs 15, 16). SP cells are also more radio-resistant and tumourigenic than their counterparts^17^. In addition, downregulation of 26S proteasome activity has been reported in CSCs from several types of solid tumours, resulting in the protection of critical regulators of CSC properties against proteasome degradation^18^,^19^.

Recently, several studies have identified small molecules that selectively reduce the number of CSCs in solid cancers^20^,^21^. Likewise, metformin, a biguanide anti-diabetic drug for T2D that originates from the herb French lilac, is able to selectively target breast CSCs^22^ and suppress tumour development in the breast, pancreas and lung^23^,^24^. In retrospective cohort studies, diabetic patients treated with metformin had a significantly lower risk of cancer mortality than those that were untreated or treated with other drugs^3^.

In our previous study, we performed a comparative genomic hybridization array analysis and identified the loss of heterozygosity on chromosome 9 (9q22.3), the locus at which miR-27b is located, in a docetaxel-resistant luminal-type human breast cancer cell line^25^. A previous clinical study also demonstrated that 9q21–22 is a putative breast cancer susceptibility locus^26^, and alternation at the 9q22.3 region is associated with early- and late-onset breast cancers caused by dysregulation of DNA repair pathways as well as the Hedgehog-dependent self-renewal pathway^27^. On the other hand, miR-27b also reportedly functions like an oncogene in breast cancer cells and is associated with poor prognosis of triple negative breast cancer patients^28^,^29^,^30^. These reports and our previous findings suggest that the functions of miR-27b are diverse and may be dependent on the specific subtype of breast cancer.

Here, we report that miR-27b inhibits the acquisition of CSC properties in luminal-type breast cancer and that metformin reduces the SP fraction of breast cancer cells through miR-27b-mediated repression of the gene ectonucleotide pyrophosphatase/phosphodiesterase family member 1 (*ENPP1*), which is involved in T2D development^31^. ENPP1 induced the generation of a SP fraction that had tumour seeding ability and was resistant to conventional chemotherapy by promoting the expression and cell surface localization of ABCG2. Growth of breast cancer cells under mammosphere culture conditions, which are used widely for the enrichment of breast CSCs^32^, induced downregulation of miR-27b and suppression of proteasome activity, leading to an accumulation of ENPP1. Furthermore, our clinical data suggest *ENPP1* expression in primary breast cancer tissues is associated with malignant potential and response to chemotherapy. Overall, the results presented here elucidate a molecular mechanism involved in the acquisition of CSC properties and demonstrate that a T2D-associated gene plays an important role in this process. Moreover, the results might contribute to current understanding of the biological link between breast cancer and T2D development.

## Results

### SP fraction is generated from miR-27b downregulated cells

**Figure 1:**
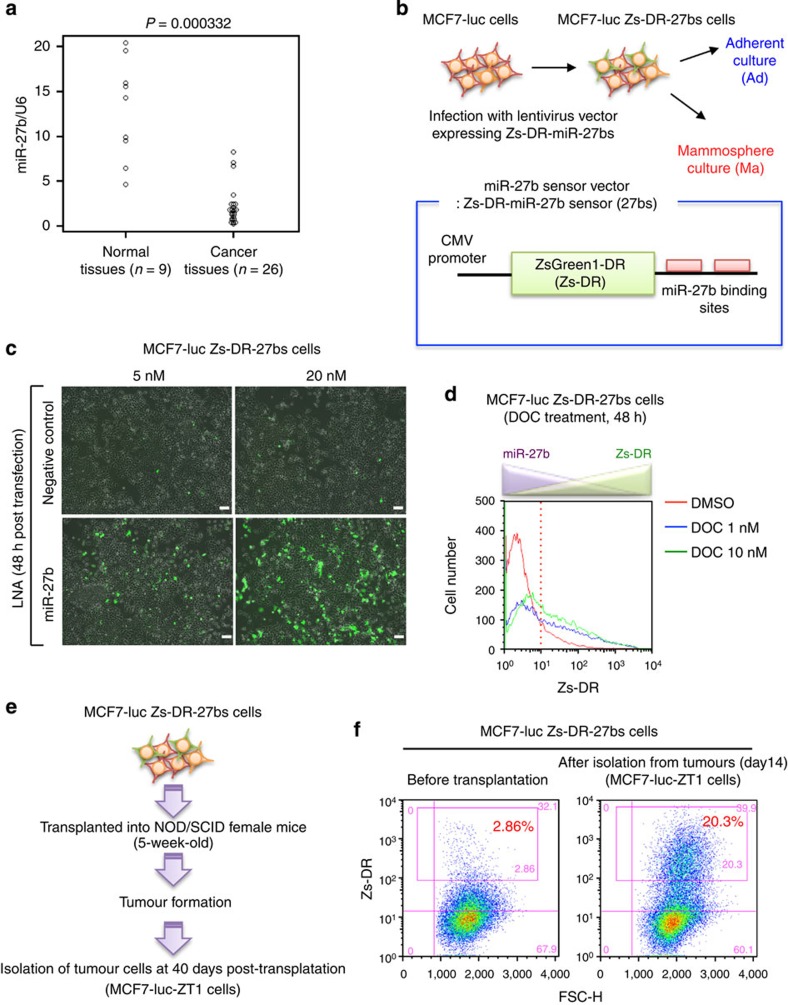
MiR-27b is involved in the drug resistance and tumorigenicity of breast cancer cells. (**a**) Relative miR-27b expression levels in normal and luminal-type breast cancer tissues. Expression levels were normalized to those of *RNU6B*. Differences between groups were analysed using unpaired *t*-tests. (**b**) Schematic illustration of the miR-27b sensor construct used in the experiments shown in **c**–**f**. (**c**) Expression of Zs-DR 48 h after transfection of MCF7-luc Zs-DR-27bs cells with a negative control or miR-27b-specific LNA. Scale bar, 100 μm. (**d**) Flow cytometric analyses of Zs-DR expression 48 h after treatment of MCF7-luc Zs-DR-27bs cells with dimethylsulphoxide (DMSO) or docetaxel (DOC). (**e**) Isolation of tumourigenic MCF7-luc Zs-DR-27bs cells. After transplantation of MCF7-luc Zs-DR-27bs cells into 5-week-old NOD/SCID mice, the cells were isolated and cultivated *in vitro*. (**f**) Flow cytometric analyses of Zs-DR expression in tumourigenic MCF7-luc Zs-DR-27bs cells isolated from the mice described in **e**.

In agreement with a previous clinical study^27^ and our finding that miR-27b is downregulated in docetaxel-resistant luminal-type breast cancer cells^25^, a quantitative reverse transcription–PCR (qRT–PCR) analysis revealed that miR-27b expression was significantly lower in luminal-type breast cancer tissues (*n*=26) than normal tissues (*n*=9; Fig. 1a). In addition, according to the data in The Cancer Genome Atlas Research Network (http://cancergenome.nih.gov/), miR-27b was significantly downregulated in the luminal-type cancer patients who received the taxane-based adjuvant chemotherapy compared with normal breast tissues (Supplementary Fig. 1a, b). Because reduced miR-27b expression was observed in both non-recurrent and recurrent patients (Supplementary Table 1), we hypothesized that downregulation of miR-27b is associated with not only drug resistance, but also tumour initiation. To test this hypothesis, we investigated the expression of miR-27b in non-drug-resistant luminal-type breast cancer MCF7 cells after docetaxel treatment and tumour formation in immunodeficient mice. The expression level of miR-27b in the parental MCF7 cell line was comparable to that in two different normal breast tissue samples (Supplementary Fig. 1c, d). To monitor miR-27b expression, we prepared the MCF7 cells that expressed the sensor vector of miR-27b^33^,^34^,^35^,^36^. After MCF7 cells expressing firefly luciferase (MCF7-luc cells) were prepared by a lentiviral vector, these cells were then transfected with a destabilized fluorescent lentiviral reporter construct (ZsGreen1-DR), whose 3′-untranslated region (3′UTR) contained two miR-27b complementary sequences, to generate MCF7-luc Zs-DR-27bs cells (Fig. 1b and Supplementary Fig. 2a, b). In this system, Zs-DR-positive cells represented those in which miR-27b was downregulated (Fig. 1b). To confirm that the expression of Zs-DR was induced by miR-27b knockdown, endogenous miR-27b expression was inhibited by transfecting the MCF7-luc Zs-DR-27bs cells with a locked nucleic acid (LNA) probe. Treatment of the cells with the miR-27b-specific LNA (LNA-miR-27b; at least 5 nM) induced Zs-DR expression in MCF7-luc Zs-DR-27bs cells, whereas treatment with a control LNA (LNA-NC) did not (Fig. 1c). Next, this cell line was used to examine the effect of docetaxel on miR-27b expression. A flow cytometry analysis revealed that docetaxel treatment induced Zs-DR expression in MCF7-luc Zs-DR-27bs cells markedly (Fig. 1d). Elevated expression of Zs-DR was also observed after tumour formation in 5-week-old non-obese diabetic/severe combined immunodeficiency (NOD/SCID) mice that were injected in the hind legs with 10^5^ MCF7-luc Zs-DR-27bs cells (Fig. 1e,f). Together with previous findings, these results suggest that downregulation of miR-27b is involved in the acquisition of docetaxel resistance and tumour seeding ability in luminal-type breast cancer cells.

**Figure 2:**
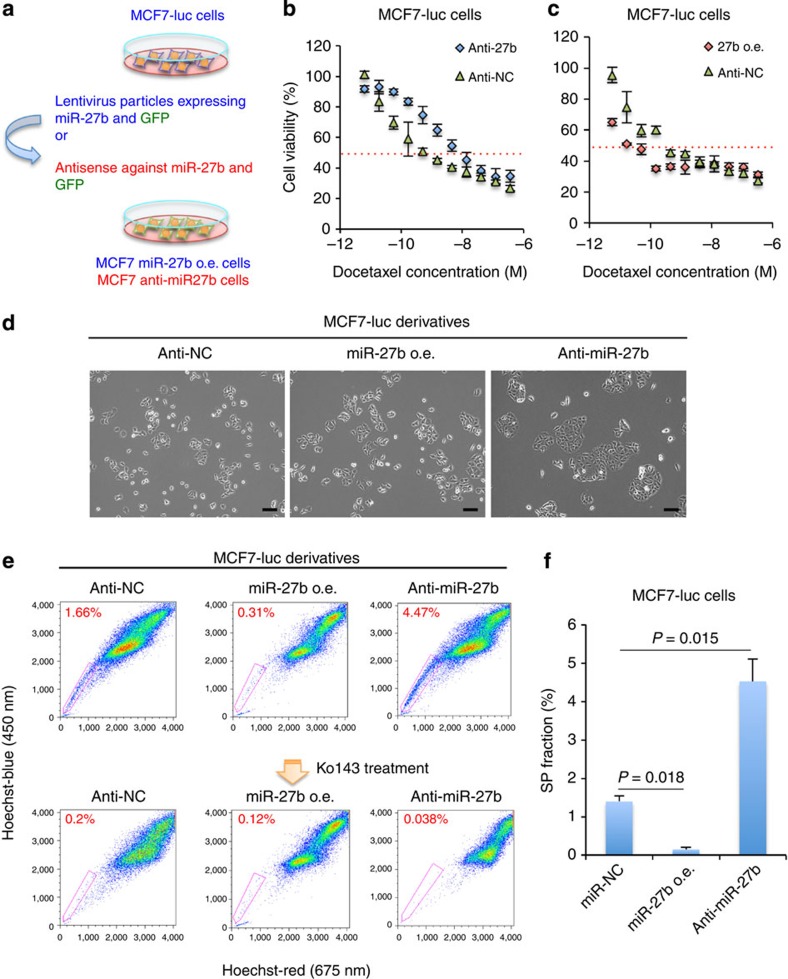
MiR-27b regulates the resistance of breast cancer cells to docetaxel. (**a**) Overview of the method used to establish miR-27b knockdown MCF7-luc (MCF7-luc anti-miR-27b) cells. (**b**,**c**) Dose–response curves of MCF7-luc anti-NC, MCF7-luc anti-miR-27b and MCF7-luc miR-27b o.e. cells treated with docetaxel. Cell viability was normalized to that of the corresponding cells treated with dimethylsulphoxide (DMSO). The red dashed line indicates the IC_50_ value. Data are represented as the mean±s.d. of *n*=3 replicates. (**d**) Morphologies of the MCF7-luc anti-NC, MCF7-luc miR-27b o.e. and MCF7-luc anti-miR-27b cells. Scale bar, 100 μm. (**e**) Flow cytometric analyses of the SP fraction of MCF7-luc derivatives in the presence and absence of Ko143. (**f**) Quantification of the SP fraction of MCF7-luc derivatives. The SP fraction was determined as the difference between the level of Hoechst 33342 staining in the presence and absence of Ko143. Data are represented as the mean±s.d. of *n*=3 replicates. Statistical significance was determined by Student's *t*-test.

To investigate its role in the regulation of CSC properties further, the effects of knockdown of miR-27b on the drug resistance and tumorigenicity of MCF7 cells were examined. A miR-27b knockdown MCF7-luc cell line (MCF7-luc anti-miR-27b) was generated using a lentiviral vector expressing an antisense miR-27b sequence (Fig. 2a). To confirm the suppression of miR-27b, MCF7-luc anti-miR-27b cells were transfected with a sensor vector (pTK-GLuc-27bs) harbouring a secreted *gaussia* luciferase reporter and two miR-27b complementary sequences in its 3′UTR (Supplementary Fig. 2a, b). A reporter assay showed that the luciferase activity in MCF7-luc anti-miR-27b cells expressing pTK-GLuc-27bs was fivefold higher than that in control cells (MCF7-luc anti-NC) expressing this reporter (Supplementary Fig. 2c). A similar assay was also performed using a luciferase reporter construct harbouring the 3′UTR of the gene encoding peroxisome proliferator-activated receptor gamma (PPARG), which has been reported as a direct target of miR-27b^37^. After transfection with the *PPARG* reporter construct, luciferase activity was significantly higher in MCF7-luc anti-miR-27b cells than control cells (Supplementary Fig. 3a, b). Overall, these results demonstrate that the lentivirus vector system inhibited the function of miR-27b efficiently.

Next, we examined the resistances of the MCF7-luc anti-NC and MCF7-luc anti-miR-27b cells to docetaxel (Fig. 2a,b). Downregulation of miR-27b in MCF7 cells induced docetaxel resistance; the IC_50_ value of docetaxel towards the MCF7-luc anti-miR-27b cells was approximately tenfold higher than that towards the MCF7-luc anti-NC cells (Fig. 2b). To confirm the role of miR-27b in acquired docetaxel resistance, a lentiviral vector was used to generate MCF7 cells overexpressing miR-27b (MCF7-luc miR-27b o.e. cells; Fig. 2a and Supplementary Fig. 3c). The IC_50_ value of docetaxel towards the MCF7-luc miR-27b o.e. cells was approximately fivefold lower than that towards the MCF7-luc anti-NC cells (Fig. 2c). Furthermore, whereas the MCF7-luc miR-27b o.e. and MCF7-luc anti-NC cells showed similar morphological phenotypes, the MCF7-luc anti-miR-27b cells were slightly larger in size (Fig. 2d). Taken together, these results demonstrate that downregulation of miR-27b induces resistance of MCF7 cells to docetaxel.

Because ABCG2 expression is related to the enhanced drug resistance of MCF7 cells^38^, the expression of this transporter in these cells was examined by flow cytometry. ABCG2 was expressed at a high level in the SP fraction of MCF7 cells (Supplementary Fig. 3d). In addition, a single-cell PCR analysis revealed downregulation of miR-27b in a few MCF7 cells (Supplementary Fig. 4a). Therefore, we hypothesized that the SP fraction was generated from cells with low miR-27b expression. In support of this theory, the SP fraction of the MCF7 anti-miR-27b cells was significantly larger than that of the MCF7 anti-NC cells, and this increase was abolished when the cells were treated with the ABCG2 inhibitor Ko143 (Fig. 2e,f). A similar result was also observed when luminal-type, non-drug-resistant breast cancer ZR75-1 cells were used (Supplementary Fig. 4b, c). Furthermore, the SP fraction of MCF7 miR-27b o.e. cells was significantly smaller than that of the control cells (Fig. 2e,f).

As knockdown of miR-27b induced docetaxel resistance in MCF7 cells, we also investigated the resistance of MCF7 cells to cisplatin. The IC_50_ value of cisplatin towards the MCF7-luc anti-miR-27b cells was slightly lower than that towards the MCF7-luc anti-NC cells (Supplementary Fig. 4d). In addition, considering the low level of expression of miR-23b in our institute cancer patients (Fig. 1a and Supplementary Fig. 5a), we examined the size of the SP fraction in miR-23b knockdown MCF7-luc cells. A flow cytometry analysis revealed that the size of the SP fraction was not affected by knockdown of miR-23b (Supplementary Fig. 5b).

**Figure 3:**
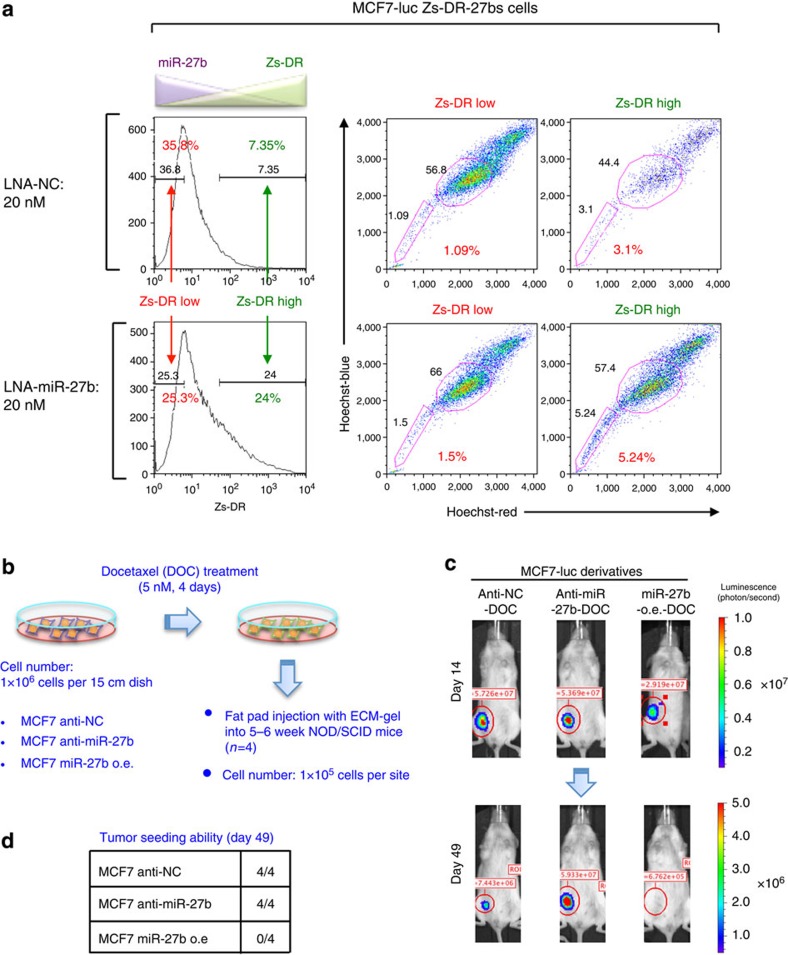
MiR-27b regulates the tumour seeding ability of breast cancer cells. (**a**) Flow cytometric analyses of MCF7-luc Zs-DR-27bs cells transfected with LNA-NC or LNA-miR-27b. (**b**) Overview of the method used to analyse the CSC properties of docetaxel-treated MCF7-luc cell derivatives. (**c**) Bioluminescent images of tumours in NOD/SCID mice injected with docetaxel (DOC)-treated MCF7-luc cell derivatives. Representative images are shown for each cohort. (**d**) The numbers of animals with detectable tumours in the groups injected with the MCF7-luc cell derivatives.

To confirm that MCF7 cells in the SP fraction show downregulation of miR-27b, the expression levels of Zs-DR and the sizes of the SP fraction were determined in MCF7-luc Zs-DR-27bs cells transfected with LNA-miR-27b or LNA-NC. In both cell types, the SP fraction of the Zs-DR-positive cells was three- to fourfold larger than that of the Zs-DR-negative cells (Fig. 3a). Notably, unlike LNA-NC, LNA-miR-27b caused a drastic increase in the number of Zs-DR-positive cells that showed the SP phenotype (Fig. 3a).

Overall, these results suggest that miR-27b attenuates drug resistance by attenuating the formation of the SP fraction of breast cancer cells. In addition, a qRT–PCR analysis revealed that miR-27b expression was lower in ZR75-1 cells than MCF7 cells (Supplementary Fig. 6a), suggesting that a significant reduction in miR-27b is essential for the acquisition of the SP phenotype.

### MiR-27b inhibits the tumour seeding ability of SP fraction

In immunodeficient mice, the SP fraction is not only resistant to anti-cancer agents, but also highly tumourigenic^17^,^39^; therefore, we investigated the tumour seeding ability of the SP fraction of MCF7 cells. Because the MCF7-luc miR-27b o.e. cells contained a relatively small SP fraction, we compared the tumour seeding abilities of these cells and MCF7-luc anti-NC cells in NOD/SCID mice (Supplementary Fig. 6b). *In vivo* imaging revealed that the MCF7-luc miR-27b o.e. cells showed lower tumour growth than the MCF7-luc anti-NC cells (Supplementary Fig. 6b). To determine whether the tumours derived from MCF7-luc cells also displayed the chemoresistance, we assessed the presence of functional CSCs by evaluating the *in vivo* tumour seeding ability of these cells after docetaxel treatment (Fig. 3b). Docetaxel-treated MCF7-luc anti-NC and MCF7-luc anti-miR-27b cells showed high tumour seeding ability in NOD/SCID mice; however, the seeding ability of docetaxel-treated MCF7-luc miR-27b o.e. cells was very low (Fig. 3c,d and Supplementary Fig. 6c). Treatment of MCF7-luc anti-miR-27b cells with docetaxel increased the SP fraction (Supplementary Fig. 6d); therefore, these results indicate the tumour seeding ability of SP fraction.

**Figure 4:**
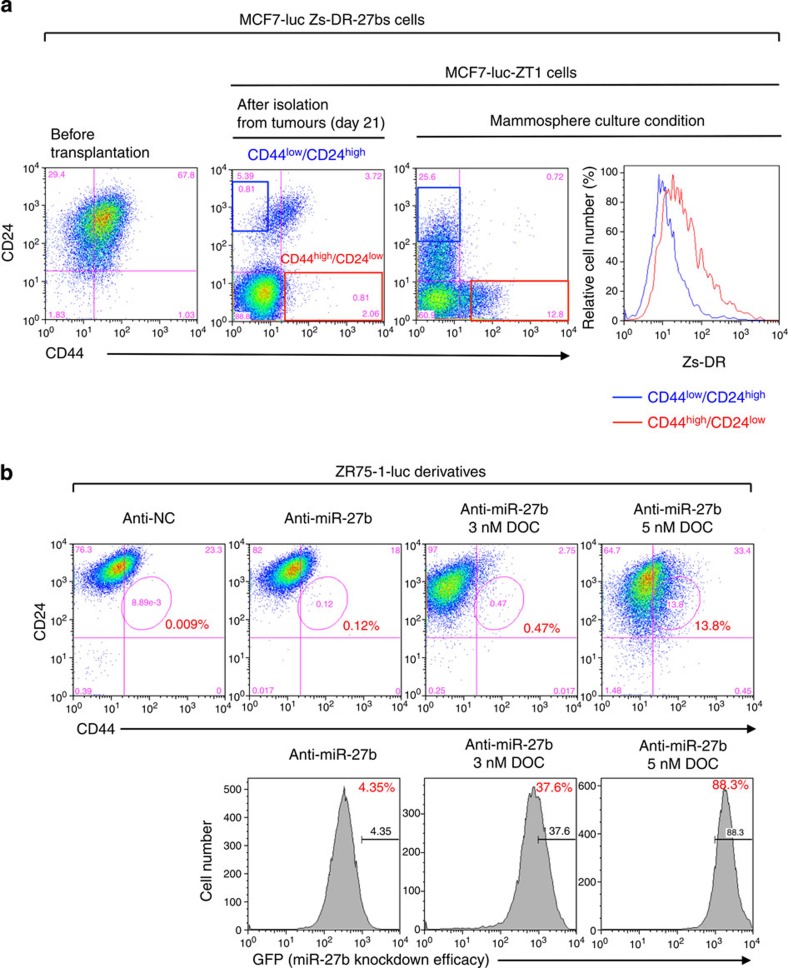
Downregulation of miR-27b is associated with the generation of CD44^high^/CD24^low^ fraction. (**a**) Flow cytometry analyses of the CD44^high^/CD24^low^ population and Zs-DR expression in MCF7-luc Zs-DR-27bs and its tumorigenic cells (MCF7-luc-ZT1 cells) in adherent and mammosphere culture conditions. (**b**) Flow cytometry analyses of the CD44^high^/CD24^low^ and GFP^high^ populations of ZR75-1-luc anti-miR-27b cells treated with docetaxel (DOC).

In breast cancer, CD44^high^/CD24^low^ cells show a higher tumour seeding ability and chemoresistance than CD44^low^/CD24^high^ cells^12^,^39^. As MCF7 cells contained the two main populations (CD44^low^/CD24^high^ and CD44^low^/CD24^low^ cell fraction), we first examined the expression of miR-27b in these two populations. A qRT-PCR analysis coupled with cell sorting revealed that miR-27b was downregulated in CD24^low^ cell fraction compared with CD24^high^ cell fraction (Supplementary Fig. 7a). We also found that CD44^low^/CD24^high^ cell fraction was decreased in MCF7-luc anti-miR-27b cells (Supplementary Fig. 7b); therefore we next investigated the role of miR-27b for the generation of CD44^high^/CD24^low^ cells using MCF7-luc-ZT1 cells (Fig. 1e,f). Because, a flow cytometry analysis revealed that approximately 90% of MCF7-luc-ZT1 cells showed CD24^low^ antigen phenotype (Fig. 4a, middle panel) and mommsphere culture conditions, which are used for the enrichment of CSCs in breast cancer cells^32^, induced the generation of CD44^high^/CD24^low^ cell fraction in MCF7-luc-ZT1 cells (Fig. 4a, right panel). Compared with CD44^low^/CD24^high^ fraction, elevated expression of Zs-DR was observed in CD44^high^/CD24^low^ fraction (Fig. 4a, right panel). Furthermore, in ZR75-1-luc anti-miR-27b cells, docetaxel treatment significantly induced the increase of CD44^high^/CD24^low^ cells (Fig. 4b, upper panel). Because the lentivirus vector expressing the miR-27b antisense sequence also expressed green fluorescent protein (GFP), we confirmed the efficiency of knockdown of miR-27b by monitoring GFP expression (Fig. 4b, lower panel). Exposure of ZR75-1-luc anti-miR-27b cells to 5 nM docetaxel induced a clear increase in the number of GFP^high^ cells (Fig. 4b, right pannel). These results suggest that miR-27b inhibits the generation of the SP fraction from the main population of cells, and that the SP fraction derived from cells with low miR-27b expression has CSC properties such as chemoresistance and high tumour seeding ability. These findings also indicate that loss or downregulation of miR-27b is essential but not sufficient for the generation of the CD44^high^/CD24^low^ fraction of luminal-type breast cancer cells.

### Identification of *ENPP1* as a direct target of miR-27b

**Figure 5:**
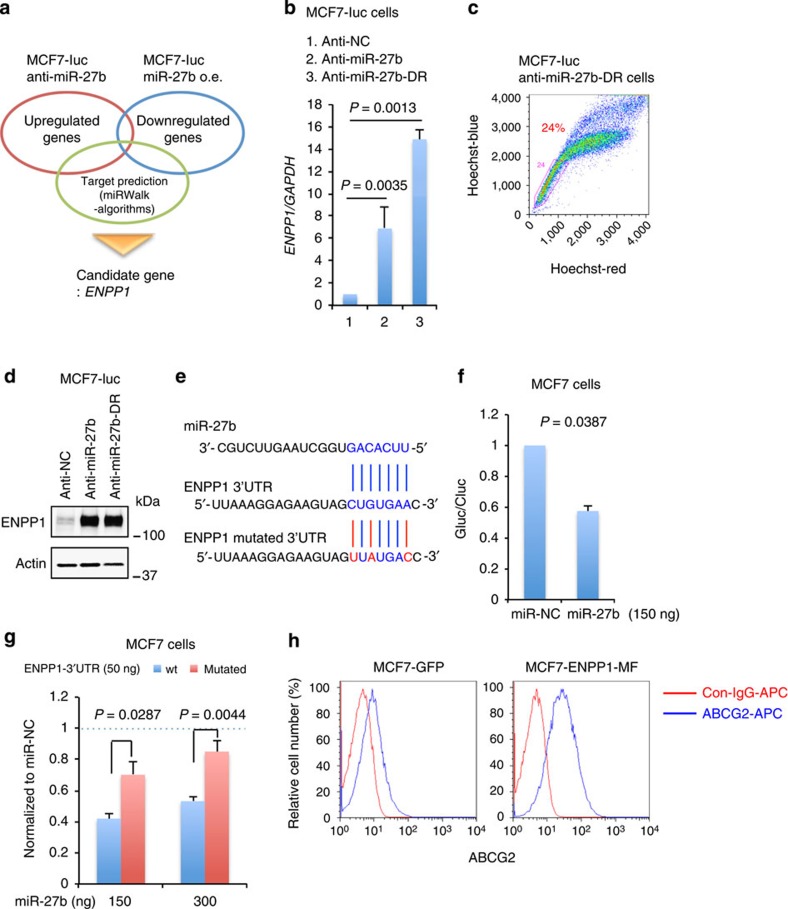
Identification of miR-27b targets. (**a**) Overview of the method used to identify potential miR-27b targets. (**b**) A qRT–PCR analysis of *ENPP1* expression in MCF7-luc derivatives. Data are represented as the mean±s.d. of *n*=3 replicates. Expression levels were normalized to those of *GAPDH*. Statistical significance was determined by Student's *t*-test. (**c**) Flow cytometric analysis of the SP fraction of MCF7-luc anti-miR-27b-DR cells. (**d**) Immunoblot analysis of ENPP1 expression in MCF7-luc derivatives. β-Actin was used as a loading control. (**e**) Sequences of miR-27b and the miR-27b-binding site in the 3′UTR of *ENPP1*. The nucleotides shown in red were mutated in the *ENPP1* 3′UTR construct used in **g**. (**f**) Luciferase activity in MCF7 cells transfected with the pTK-GLuc reporter construct containing the wild-type 3′UTR of *ENPP1* (50 ng), a miR-27b or nonspecific miRNA (miR-NC) expression vector and the pSV40-cLuc vector (50 ng). The ratio of *Gaussia* to *Cypridina* luciferase activity (GLuc/CLuc) was determined. Data are represented as the mean±s.d. of *n*=3 replicates. The *P*-values were calculated by Student's *t*-test. (**g**) Luciferase activity in MCF7 cells transfected with pTK-GLuc containing the wild-type (wt) or mutant 3′UTR of *ENPP1* (50 ng), a miR-27b or nonspecific miRNA (miR-NC) expression vector, and pSV40-cLuc (50 ng). Data were normalized to luciferase activity in the corresponding cells transfected with miR-NC and are represented as the mean±s.d. of *n*=3 replicates. The *P*-values were calculated by Student's *t*-test. (**h**) Flow cytometric analysis of ABCG2 expression in MCF7-GFP cells and MCF7-luc cells transfected with the ENPP1-MF expression vector. The red and blue lines indicate the results of control IgG-APC and ABCG2-APC, respectively.

To identify the target of miR-27b that induces the generation of the SP fraction, a microarray analysis was combined with the use of miRNA target-predicting algorithms (miRWalk)^40^. Candidate miR-27b target genes that were upregulated (>2-fold, *P*<0.05) in MCF7-luc anti-miR-27b cells and downregulated in MCF7-luc miR-27b o.e. cells (<0.7-fold, *P*<0.05) compared with MCF7-luc anti-NC cells were identified (Fig. 5a and Supplementary Table 2). Among the candidates identified, we selected genes containing a miR-27b-binding site in their 3′UTR. Among these genes, *ENPP1* showed the most drastic difference in expression between MCF7-luc anti-miR-27b and MCF7-luc miR-27b o.e. cells (Supplementary Table 2). A qRT–PCR analysis coupled with cell sorting also revealed elevated expression of the *ENPP1* and *ABCG2* mRNAs in the SP fraction of MCF7 cells (Supplementary Fig. 8a–c). Consistent with these results, a qRT–PCR analysis revealed that *ENPP1* expression in MCF7-luc anti-miR-27b cells was approximately sixfold higher than that in MCF7-luc anti-NC cells (Fig. 5b). Next, we established a docetaxel-resistant derivative of the MCF7-luc anti-miR-27b cell line (MCF7-luc anti-miR-27b-DR) by stepwise exposure to docetaxel. *ENPP1* expression was even higher (approximately 15-fold) in MCF7-luc anti-miR-27b-DR cells, which had a large SP fraction (Fig. 5b,c). In addition, an immunoblot analysis revealed that ENPP1 was expressed at a higher level in MCF7-luc anti-miR-27b and anti-miR-27b-DR cells than MCF7-luc anti-NC cells (Fig. 5d).

To determine whether *ENPP1* is a direct target of miR-27b, MCF7 cells were co-transfected with an expression vector containing miR-27b or a nonspecific control miRNA, and a luciferase reporter vector harbouring the 3′UTR of *ENPP1* containing the wild-type or mutated miR-27b-binding site (Fig. 5e). For this experiment, we selected the one candidate site in the 3′UTR of *ENPP1* that several algorithms commonly selected as the target site of miR-27b in miRWalk. As expected, luciferase activity in cells transfected with the miR-27b expression vector was lower than that in cells transfected with the negative control miRNA (Fig. 5f). In addition, luciferase expression was suppressed to a greater extent by miR-27b in cells transfected with the wild-type *ENPP1* construct than those transfected with the mutated construct (Fig. 5g). In a similar experiment, the luciferase activity of the *ENPP1* construct was induced significantly by co-transfection of MCF7 cells with an expression vector containing a miR-27b antisense sequence (Supplementary Fig. 8d). Overall, these results suggest that miR-27b regulates *ENPP1* directly by binding to a target site in its 3′UTR.

Next, the role of ENPP1 in the acquisition of CSC properties was investigated. Gene expression analyses of MCF7-luc cells transiently transfected with an expression vector harbouring C-terminally Myc- and Flag-tagged ENPP1 (MCF7-ENPP1-MF) were performed (Supplementary Table 3). An enlarged SP fraction was one of the important phenotypes of miR-27b knockdown cells, and ABC transporters are mainly responsible for the SP phenotype^14^,^39^; therefore, we focused on the expression of ABCG2, which was commonly upregulated in MCF7-luc anti-miR-27b and MCF7-ENPP1-MF cells. Compared with that in control (MCF7-luc anti-NC) cells, expression of the *ABCG2* gene was at least twofold higher in both test cell lines (Supplementary Table 3). A flow cytometric analysis showed that ENPP1 also promoted the cell surface localization of ABCG2 in MCF7 cells (Fig. 5h). These results suggest that miR-27b inhibits the expression and cell surface localization of ABCG2 by suppressing *ENPP1*, leading to a reduced SP fraction.

### ENPP1 promotes the cell surface localization of ABCG2

**Figure 6:**
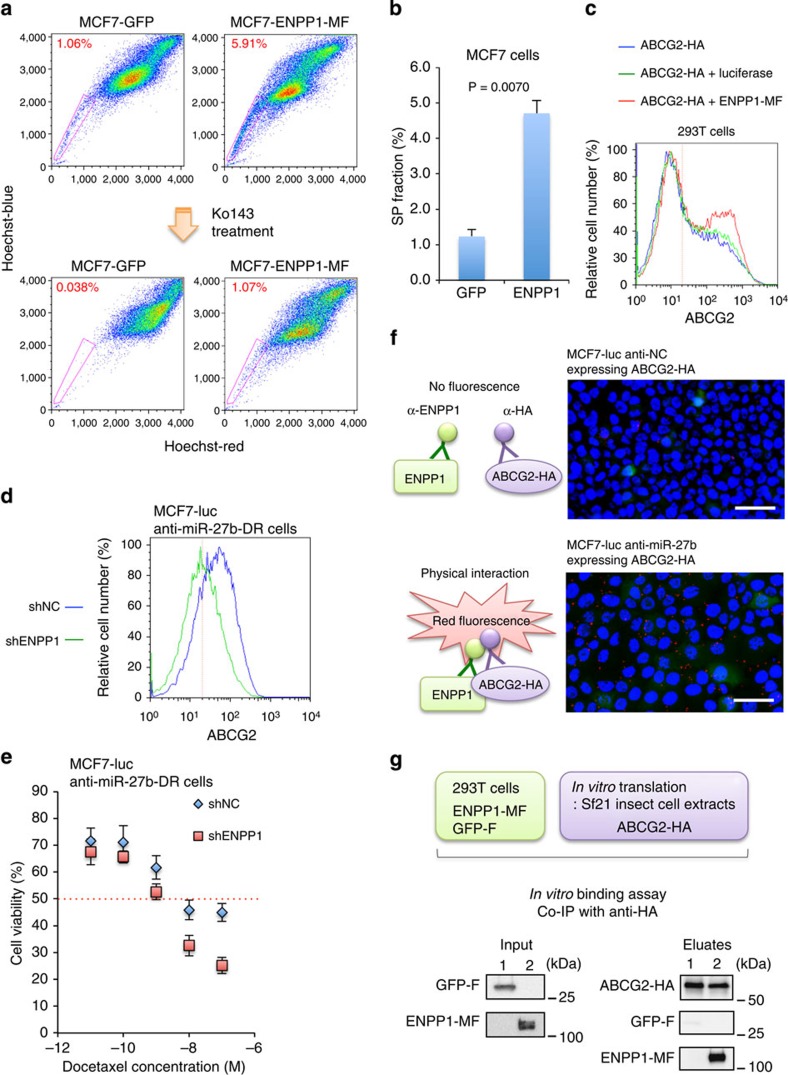
Functional analysis of ENPP1 in MCF7-luc cells. (**a**) Flow cytometric analysis of the SP fractions of MCF7-luc cells overexpressing ENPP1-MF or GFP as a control, in the presence and absence of Ko143. (**b**) Quantification of the SP fractions shown in **a**, determined as the difference between the level of Hoechst 33342 staining in the presence and absence of Ko143. Data are represented as the mean±s.d. of *n*=3 replicates. (**c**) Flow cytometric analysis showing the cell surface localization of ABCG2 in the indicated 293T co-transfectants. (**d**) Flow cytometric analyses of the cell surface localization of ABCG2 in MCF7-luc anti-miR-27b cells transfected with a control (shNC) or ENPP1-specific (shENPP1) shRNA. (**e**) Dose–response curves of docetaxel-treated MCF7-luc anti-miR-27b-DR cells transfected with shNC or shENPP1. Cell viability was normalized to that of the corresponding cells treated with dimethylsulphoxide (DMSO). The red dashed line indicates the IC_50_ value. Data are represented as the mean±s.d. of *n*=3 replicates. (**f**) Proximity ligation assay using MCF7-luc anti-NC or MCF7-luc anti-miR-27b cells transiently expressing ABCG2-HA. Scale bar, 50 μm. (**g**) *In vitro* binding assay using C-terminally Flag-tagged GFP or C-terminally Myc- and Flag-tagged ENPP1 purified from 293T cells and C-terminally HA-tagged ABCG2 purified from Sf21 insect cell extracts.

To confirm that ENPP1 induces generation of the SP fraction of MCF7 cells by regulating the expression and cell surface localization of ABCG2, a flow cytometric analysis of MCF7-ENPP1-MF cells was performed (Fig. 6a and Supplementary Fig. 8e). As expected, the SP fraction of the MCF7-ENPP1-MF cells was approximately fivefold larger than that of control cells transfected with a vector expressing GFP, and this increase was abolished after treatment with Ko143 (Fig. 6a,b). Because ENPP1 is not a transcription factor and localizes mainly to the cytoplasm and cell membrane^41^, we hypothesized that ENPP1 induces ABCG2 expression indirectly. Therefore, we performed a flow cytometric analysis using 293T cells overexpressing C-terminally HA-tagged ABCG2 (ABCG2-HA) and ENPP1-MF or firefly luciferase as a control. Overexpression of ENPP1-MF enhanced the cell surface localization of ABCG2 in 293T cells markedly (Fig. 6c). Next, we knocked down ENPP1 in MCF7-luc anti-miR-27b-DR cells using a lentivirus vector (Supplementary Fig. 8f) and examined its effects on the cell surface localization of ABCG2. A flow cytometry analysis revealed that knockdown of ENPP1 inhibited the cell surface localization of ABCG2 in these cells drastically (Fig. 6d). In addition, knockdown of ENPP1 reduced the docetaxel resistance of MCF7-luc anti-miR-27b-DR cells (Fig. 6e).

To examine the physical interaction between ENPP1 and ABCG2, proximity ligation and *in vitro* binding assays were performed. The proximity ligation assay revealed that the interaction of ENPP1 with ABCG2 was significantly higher in MCF7-luc anti-miR-27b cells transiently expressing ABCG2-HA than MCF7-luc anti-NC cells transiently expressing ABCG2-HA (Fig. 6f). Furthermore, the *in vitro* binding assay using ENPP1-MF purified from 293T cells and ABCG2-HA purified from Sf21 insect cell extracts suggested a direct physical interaction of ENPP1 with ABCG2 in MCF7-luc anti-miR-27b cells (Fig. 6g).

According to a recent structural mapping study, human ENPP1 comprises a cytosolic, catalytic and nuclease domain^41^. To determine the domain involved in binding of ENPP1 to ABCG2, co-immunoprecipitation analyses were performed using extracts from 293T cells expressing ABCG2-HA and ENPP1 deletion mutants (Supplementary Fig. 8g and 9a–c). These experiments revealed that the N-terminal region containing the cytosolic domain of ENPP1 is critical for the interaction with ABCG2 (Fig. 6f,g and Supplementary Fig. 9). Because upregulation of miR-27b reduced the tumourigenic SP fraction (Fig. 3b–d) and ENPP1 was expressed at a high level in this fraction (Supplementary Fig. 8a–c), these results indicate that ENPP1 contributes to the acquisition of resistance to anti-cancer agents.

### ENPP1 is a target for the proteasome pathway

**Figure 7:**
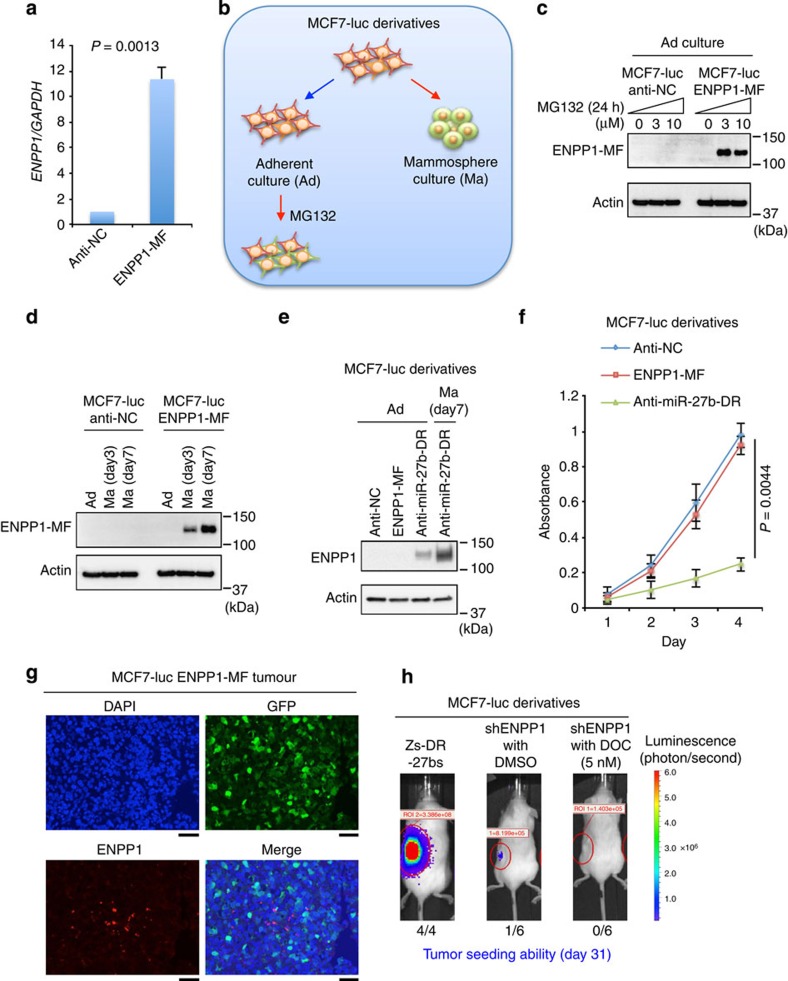
ENPP1 is a substrate of the 26S proteasome. (**a**) *ENPP1* expression in MCF7-luc cells stably expressing ENPP1-MF or anti-NC as a control, detected by qRT–PCR. Expression levels were normalized to those of *GAPDH* and data are represented as the mean±s.d. of *n*=3 replicates. The *P*-values were calculated by Student's *t*-test. (**b**) A schematic illustration of the approach used in the experiments shown in **c**–**e**. (**c**) Immunoblot analyses of ENPP1 expression in MCF7-luc anti-NC and MCF7-luc ENPP1-MF cells treated with or without MG132 for 24 h. (**d**) Immunoblot analyses of ENPP1 in MCF7-luc ENPP1-MF cells grown under adherent (Ad) or mammosphere (Ma) culture conditions for the indicated times. (**e**) ENPP1 expression in the indicated MCF7-luc derivatives grown under adherent (Ad) or mammosphere (Ma) culture conditions. (**f**) Growth of the indicated MCF7-luc cell derivatives. An MTT assay was performed to determine the numbers of cells at each time point. Data are represented as the mean±s.d. of *n*=3 replicates. The *P*-values were calculated by Student's *t*-test. (**g**) Immunofluorescent detection of ENPP1-MF and GFP in MCF7-luc ENPP1-MF cells in paraffin-embedded sections of primary tumour xenografts. The nuclei were stained with 4,6-diamidino-2-phenylindole (DAPI). Scale bar, 50 μm. (**h**) Bioluminescent images of tumours in NOD/SCID mice injected with MCF7-luc shENPP1 cells that were treated with or without docetaxel (DOC). Alternatively, the mice were injected with MCF7-luc Zs-DR-27bs cells as a technical control. Representative images are shown for each cohort.

To determine the molecular mechanism by which ENPP1 induces drug resistance in only a small population of breast cancer cells, we investigated the fate of this protein in MCF7-luc cells stably expressing ENPP1-MF (MCF7-luc ENPP-MF), which was generated using a lentivirus vector. Because the lentivirus vector expressing ENPP1 also expressed GFP, a polyclonal cell line was established by flow cytometric sorting of GFP-positive cells. The expression of ENPP1-MF in stable transfectants was confirmed by immunoblotting at day 7 after infection (Supplementary Fig. 10a). However, after 2 weeks of culture, ENPP1-MF expression in these cells was reduced markedly. At the same time point, *ENPP1* mRNA expression was tenfold higher in the MCF7-luc ENPP1-MF cells than the control cells (MCF7-luc anti-NC; Fig. 7a), suggesting that the stability of ENPP1 is affected by post-translational modifications.

Some key molecules for stem cell maintenance, such as BMI1, OCT4 and NANOG, are substrates for the 26S proteasome, and downregulation of proteasome activity is associated with the acquisition of CSC properties^42^,^43^,^44^. Therefore, we hypothesized that ENPP1 is degraded by the 26S proteasome in breast cancer cells. To test this hypothesis, the effect of the 26S proteasome inhibitor MG132 on ENPP1 expression in MCF7-luc ENPP1-MF cells was examined (Fig. 7b,c). An immunoblot analysis revealed that ENPP1-MF expression was enhanced after treatment of the cells with 3 or 10 μM MG132 (Fig. 7c).

Downregulation of proteasome activity and CSC enrichment occur under mammosphere culture conditions^18^ (Fig. 4a); therefore, the accumulation of ENPP1-MF in MCF7-luc ENPP1-MF cells cultured under these conditions was examined (Fig. 7b). Under the same conditions, we confirmed that miR-27b was downregulated and ALDH was upregulated in MCF7-luc Zs-DR-27bs cells (Supplementary Fig. 10b, c). As expected, ENPP1-MF expression was observed after 3 or 7 days of mammosphere culture (Fig. 7d). Overall, these results demonstrate that the stability of ENPP1 is regulated by the 26S proteasome and its expression is restricted to the SP fraction.

Whereas ENPP1 was accumulated in MCF7-luc ENPP1-MF cells after MG132 treatment, as mentioned above, ENPP1 expression was also detected in MCF7-luc anti-miR-27b and MCF7-luc anti-miR-27b-DR cells in the absence of MG132 (Figs 5d and 7e). The qRT–PCR analyses also confirmed that *ENPP1* was expressed at an approximately twofold higher level in MCF7-luc anti-miR-27b-DR cells than MCF7-luc ENPP1-MF cells (compare Figs 5b and 7a). Because proteasome activity is downregulated in dormant or slow cycling MCF7 cells^18^, we hypothesized that the stability of ENPP1 is dependent on the cell proliferation rate. In support of this theory, a cell proliferation assay revealed that, while there was little difference between the growth rates of MCF7-luc anti-NC cells and MCF7-luc ENPP1-MF cells, the growth rate of MCF7-luc anti-miR-27b-DR cells was significantly slower than that of MCF7-luc ENPP1-MF cells (Fig. 7f).

To investigate the effect of ENPP1 expression on tumour development further, MCF7-luc ENPP1-MF cells were subcutaneously injected into three NOD/SCID mice. *In vivo* imaging revealed that tumour development occurred in all three mice (Supplementary Fig. 11a) and ENPP1 expression was detected in approximately 5% of the tumour cells (Fig. 7g). Because the MCF7-luc ENPP1-MF cells also expressed GFP, we confirmed that the tumour tissues were derived from MCF7-luc ENPP1-MF cells by detecting GFP expression (Fig. 7g). As MCF7-luc anti-miR-27b and MCF7-luc anti-miR-27b-DR cells showed ENPP1 expression even under adherent culture conditions (Figs 5 and 7), these results suggest that the expression and function of ENPP1 is restricted to conditions in which miR-27b is lost or downregulated.

Next, we examined the tumour seeding ability of ENPP1 knockdown MCF7-luc cells (MCF7-luc shENPP1 cells) using 5-week-old NOD/SCID mice (Fig. 7h). Whereas MCF7-luc Zs-DR-27bs cells showed high tumour seeding ability in NOD/SCID mice at day 31 after subcutaneous injection, the seeding ability of dimethylsulphoxide or docetaxel-treated MCF7-luc shENPP1 cells was very low (Fig. 7h and Supplementary Fig. 11b). Taken together, these results indicate that ENPP1 expression is induced during anchorage-dependent growth and is associated with tumour formation. Based on these findings, we propose that ENPP1 expression is regulated by two distinct molecular mechanisms at the mRNA and protein levels, and that the phenotypes of the SP fraction, such as drug resistance and high tumourigenicity, are observed in a small population of cells that display downregulation of miR-27b and low proteasome activity. Because miRNAs have multiple targets^45^, other targets of miR-27b may also be involved in the cell cycle regulation of breast cancer cells.

### ENPP1 is a target of metformin

**Figure 8:**
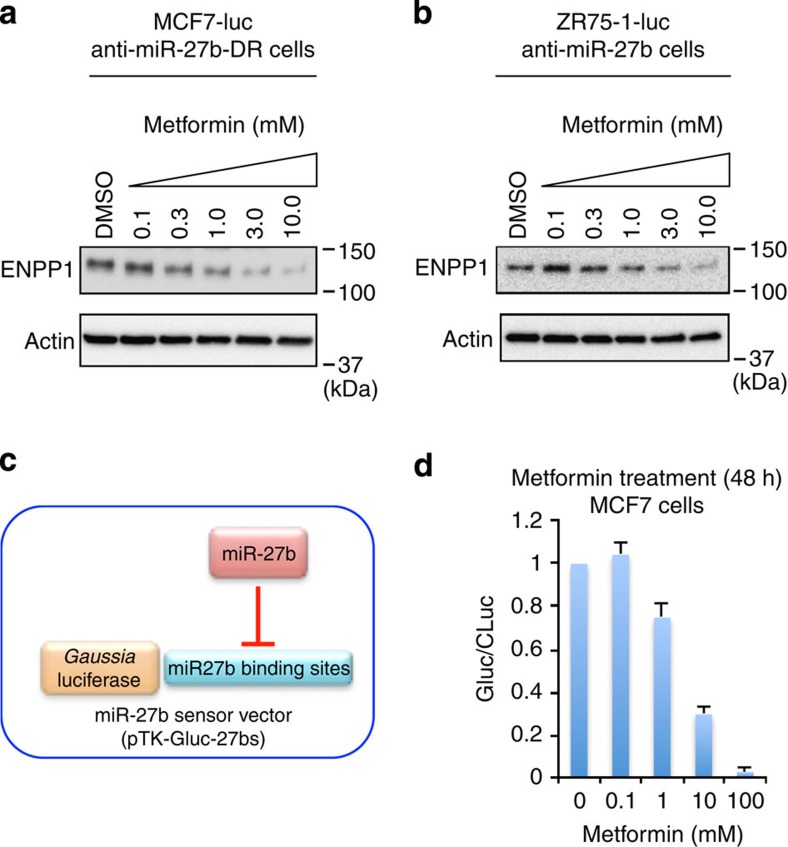
Metformin induces miR-27b-mediated suppression of ENPP1. (**a**,**b**) Immunoblot analyses of ENPP1 expression in MCF7-luc anti-miR-27b-DR (**a**) and ZR75-1-luc anti-miR-27b (**b**) cells incubated with metformin (0.1–10 mM) for 72 h. β-Actin was used as a loading control. (**c**) A schematic illustration of the miR-27b sensor construct used in the experiment shown in **d**. (**d**) Luciferase activity in MCF7 cells co-transfected with pTK-GLuc-27bs (50 ng) and pSV40-CLuc (50 ng), and then incubated with metformin for 48 h. The ratio of *Gaussia* to *Cypridina* luciferase activity (GLuc/CLuc) was determined. Data are represented as the mean±s.d. of *n*=3 replicates.

Because metformin reduces the SP fraction of breast cancer cells^46^ and generation of the SP fraction was induced by ENPP1, we hypothesized that metformin attenuates the chemoresistance of the SP fraction through miR-27b-mediated suppression of *ENPP1*. To test this hypothesis, ENPP1 expression was examined in MCF7-luc anti-miR-27b-DR and ZR75-1-luc anti-miR-27b cells that were incubated with 0.1–10 mM metformin for 72 h. Immunoblotting revealed a dose-dependent suppression of ENPP1 expression by metformin in both cell lines (Fig. 8a,b and Supplementary Fig. 12a). In addition, analysis of miR-27b expression in metformin-treated MCF7 cells using the miR-27b sensor vector (pTK-GLuc-27bs; Fig. 8c and Supplementary Fig. 2) revealed a dose-dependent suppression of luciferase activity (Fig. 8d). We also confirmed that metformin treatment reduced the SP fraction in MCF7 cells with the upregulation of miR-27b (Supplementary Fig. 12b, c). These results suggest that ENPP1 is a novel target of metformin in breast cancer cells and that metformin attenuates drug resistance through miR-27b-mediated suppression of *ENPP1*.

### ENPP1 expression is elevated in breast cancer specimens

**Figure 9:**
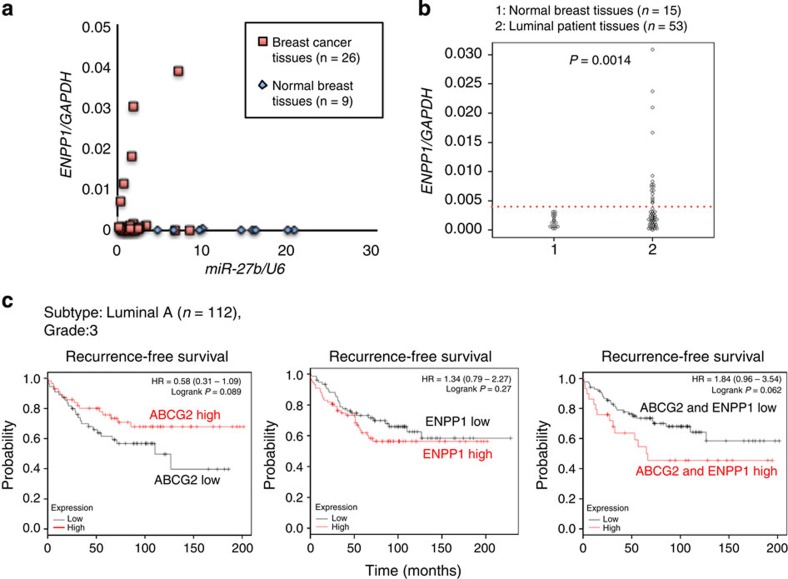
Evaluation of *ENPP1* expression in breast cancer specimens. (**a**) Evaluation of *ENPP1* mRNA expression in the clinical samples. The *y* axis displays the expression level of *ENPP1* relative to that of *GAPDH* and the *x* axis displays the expression level of miR-27b normalized to that of *RNU6B*. (**b**) Evaluation of *ENPP1* mRNA expression in breast cancer clinical samples classified by luminal-type. Expression levels were normalized to those of *GAPDH*. Comparisons between groups were performed using unpaired *t*-tests. (**c**) Kaplan–Meier representations of the probabilities of recurrence-free survival in 112 breast cancer cases classified according to the expression levels of *ABCG2* and *ENPP1*. The *P*-values were calculated using log rank tests.

Finally, the diagnostic value of *ENPP1* expression was evaluated by a qRT–PCR analysis of a subset of primary human breast cancer specimens. As shown in Fig. 1a, miR-27b expression was low in a subset of the tumour specimens; therefore, *ENPP1* expression was determined in these 26 samples and 9 non-tumour controls. A qRT–PCR analysis revealed elevated *ENPP1* expression in breast cancer tissues that had lower miR-27b expression than normal tissues (Fig. 9a). *ENPP1* mRNA expression was also significantly higher in 53 human primary luminal-type breast tumours than 15 normal controls (Fig. 9b). In addition, we evaluated the prognostic value of *ABCG2* and *ENPP1* expression in a public clinical microarray database of breast tumours from 112 luminal A patients (tumour grade 3)^47^. Although elevated expression of *ABCG2* or *ENPP1* was not correlated with recurrence of breast cancer, co-expression of *ABCG2* and *ENPP1* was moderately associated with poor prognosis (HR=1.84; *P*=0.062; Fig. 9c).

To confirm the prognostic values of *ENPP1* and *ABCG2* expression in other types of cancer, we examined the expression levels of these mRNAs in luminal B and basal-type breast cancer samples. Although co-expression of *ENPP1* and *ABCG2* was not correlated significantly with poor prognosis of luminal B patients, the tendency was similar to that observed for luminal A patients (Fig. 9 and Supplementary Fig. 13a). On the other hand, a correlation between poor prognosis and the co-expression of *ENPP1* and *ABCG2* was not observed for basal-type breast cancer patients (Supplementary Fig. 13b). In addition, in the triple negative (basal-type) breast cancer cell line MDA-MB-231, which shows low expression of endogenous ABCG2, an inverse correlation between *ENPP1* and miR-27b expression was observed (Supplementary Fig. 13c, d), although the cell surface localization of ABCG2 was not induced by knockdown of miR-27b (Supplementary Fig. 13e).

A recent study reported that ABCG2 is a promising marker of luminal progenitor cells in BRCA^−^ breast cancer cells^48^; hence, the results described above indicate that ENPP1 plays an important role in the acquisition of CSC properties, mainly in luminal-type or ABCG2-expressing breast cancer cells. This hypothesis is currently being investigated using breast cancer tissues and an early analysis revealed that, unlike breast cancer tissues that display elevated expression of ENPP1, this protein was expressed mainly in a small part of mammary ductal epithelial cells in adjacent normal breast tissues (Supplementary Fig. 14). As the expression and accumulation of ENPP1 were observed only in cells that showed downregulation of miR-27b and low proteasome activity (Figs 5 and 7), these results indicate that miR-27b and *ENPP1* expression may be useful early diagnostic markers to predict the malignant potential of luminal-type breast cancers.

## Discussion

Although CSCs and their specific markers have been identified in various types of cancer, the molecular mechanisms by which cells acquire CSC properties such as drug resistance and tumour seeding ability are not fully understood. Here, we demonstrate that miR-27b regulates the generation of an SP fraction that shows docetaxel resistance and high tumourigenicity. The gene encoding ENPP1, a negative modulator of insulin receptor activation^49^,^50^ that is associated with T2D development^31^, was identified as a direct target of miR-27b. ENPP1 induced the generation of the SP fraction by promoting the expression and cell surface localization of ABCG2. In addition, mammosphere culture conditions that enrich breast CSCs^32^ induced downregulation of miR-27b and suppression of proteasome activity, which was required for the induction and stabilization of ENPP1 expression. Furthermore, the anti-T2D drug metformin inhibited the generation of the SP fraction through miR-27b-mediated suppression of *ENPP1*.

Drug resistance is a major cause of cancer recurrence. A relapse phenotype may be acquired via therapy-dependent selection of a resistant minor population or adaptation of the original tumour cells to cancer treatment. The ABC transporters ABCB1, ABCC1 and ABCG2 play crucial roles in the development of multidrug resistance^17^,^51^,^52^. In several tumours, chemotherapy-resistant cells are more tumourigenic than their parental cells^14^,^53^; therefore, if the drug-resistant subpopulation can act as a major driver of tumour initiation and mediate universal therapeutic resistance, approaches that target this phenotype could improve the efficacy of treatment regimens and reduce the risk of cancer relapse. Because multidrug resistance involves a complex interplay between a number of important cellular pathways that are fundamental to the protection and survival of both normal and malignant cells^54^,^55^, it will be necessary to develop approaches for tumour-specific suppression of ABC transporters. Here, downregulation of miR-27b was observed in breast cancer patients and upregulation of miR-27b expression induced a selective reduction of the SP fraction that displayed drug resistance and high tumourigenic activity. Therefore, modulation of miR-27b expression in breast cancer tissues may be a promising approach to prevent the acquisition of a therapy-resistant phenotype.

*ENPP1* was identified as a target of miR-27b that controls the efflux activity of ABCG2 by regulating its expression and cell surface localization. The cytosolic domain of ENPP1 was essential for interaction with ABCG2. ENPP1 is a member of the ectonucleotide pyrophosphatase/phosphodiesterase family of proteins, which are conserved in vertebrates and hydrolyse pyrophosphate or phosphodiester bonds in various extracellular compounds, such as nucleotides and lysophospholipids^41^. ENPP1 also induces insulin resistance via a physical interaction with the insulin receptor^49^ and increases the risk of glucose intolerance and T2D^31^. The results presented here demonstrate that ENPP1 also regulates the drug resistance of the SP fraction in breast cancer. In addition, since elevated expression of *ENPP1* was observed in breast cancer patients, evaluation of *ENPP1* expression in primary breast cancer tissues may be useful for predicting malignant potential and response to chemotherapy.

Accumulating lines of evidence suggest that diabetes is associated with the incidence and mortality of a number of malignancies, such as colon, pancreatic and breast cancers^3^; hence, the results presented here might also contribute to current understanding of the biological link between breast cancer and T2D development. It should be noted that we could not elucidate the molecular mechanism by which ENPP1 induces upregulation of *ABCG2* at the mRNA level or the role of ENPP1 in the acquisition of tumour seeding ability; therefore, additional investigations of ENNP1 functions are required.

ENPP1 was also identified as a novel target of metformin-induced miR-27b. Metformin downregulates the mammalian target of rapamycin pathway, which is frequently mutated and dysregulated in cancer cells, especially in CSCs including the SP fraction^56^. A recent study reported that metformin selectively blocks the inflammatory pathway in breast CSCs by inhibiting nuclear factor-κB nuclear localization and signal transducers and activators of transcription 3 activity, resulting in the suppression of breast CSC phenotypes such as the CD44^high^/CD24^low^ antigen phenotype^6^. However, the molecular mechanism by which metformin restores the chemosensitivity of CSCs is still poorly understood. Therefore, the results presented here may represent a novel molecular mechanism for the anti-CSC effects of metformin. A high concentration of metformin was required to upregulate miR-27b in breast cancer cells; therefore, it may be necessary to identify other small compounds that specifically regulate miR-27b expression.

Proteasome-mediated degradation is a key post-translational modification involved in the regulation of stem cell pluripotency and differentiation^57^. Stem cell factors such as NANOG and OCT4, as well as effector proteins involved in Hedgehog signalling, are substrates of the 26S proteasome^44^,^58^,^59^, and downregulation of the 26S proteasome activity is important for the maintenance of CSCs^19^. A recent study reported downregulation of 26S proteasome activity during mammosphere culture of breast cancer cells, which led to the acquisition of CSC properties^18^. Here, downregulation of miR-27b was observed during growth of breast cancer cells under mammosphere culture conditions, and the expression and stabilization of ENPP1 was also enhanced under these conditions and during tumour formation, suggesting that key regulators of CSC properties may be controlled by two independent post-transcriptional mechanisms.

In conclusion, this study has implications for the treatment of breast cancer and might contribute to current understanding of the biological link between breast cancer initiation and T2D development. The positive effects of miR-27b in attenuating chemoresistance and tumour seeding ability suggest that, alongside conventional chemotherapy, modulation of miR-27b expression by RNA-based treatments may improve therapeutic outcomes of breast cancer patients.

## Methods

### Antibodies

The primary antibodies and dilutions were as follows: anti-ENPP1 (1:1,000; sc-393419, Santa Cruz Biotechnology), anti-ALDH (1:1,000; 611194, BD Biosciences), anti-HA (1:2,000; #3724, CST), anti-Flag (1:5,000; M185-7, MBL) and anti-actin (1:5,000; PM053-7, MBL). For co-immunoprecipitation, anti-Flag agarose (3,325, MBL) and anti-HA agarose (MBL, 3,320, MBL) were used. Immunoblotting was preformed according to the standard procedures using the primary antibodies described above. Original data of immunoblotting in the figures are presented in Supplementary Figs 12 and 15. Staining was performed using Alexa 488 or Alexa 594 (Molecular Probes) and immunofluorescence-stained cells were observed by fluorescence microscopy (BZ-X700, Keyence).

### Plasmids

The expression vectors containing human miR-27b and its complementary sequence were purchased from System Biosciences. To generate the miR-27b sensor plasmid, tandemly repeated miR-27b-binding sites were cloned into the 3′UTR of a reporter gene encoding destabilized ZsGreen1 (ZsGreen1-DR) driven by the cytomegalovirus (CMV) promoter, or secreted *Gaussia* luciferase (GLuc) driven by the TK promoter (NEB). A 500-bp fragment of the wild-type 3′UTR of human *ENPP1* encompassing the miR-27b-binding site was obtained by PCR from MCF7 genomic DNA and cloned into the pTK-GLuc vector to generate pTK-GLuc-wt-ENPP1-3′UTR. The mutant *ENPP1* 3′UTR construct (pTK-GLuc-mt-ENPP1-3′UTR) was generated by site-directed mutagenesis. The ENPP1-Myc-Flag and ABCG2-Myc-Flag expression vectors were purchased from Origene. To prepare the ABCG2-HA expression vector (pCMV6-ABCG2-HA), the *Sgf*I/*Xho*I digested fragment of the *ABCG2* cDNA and oligonucleotides primers for the HA-tag were inserted into pCMV6-AC (Origene). To prepare the lentiviral vector expressing ENPP1-Myc-Flag, the ENPP1-Myc-Flag PCR fragment was inserted into pCDH-CMV-MCS-EF1-Greenpuro (System Biosciences). A luciferase reporter construct containing the 3′UTR of *PPARG* was purchased from Genecopoeia. The sequences of the sensor vectors, LNA probes and primers used in this study are listed in Supplementary Table 4.

### Lentiviral short hairpin RNA (shRNA) transduction

Cell lines stably expressing the ENPP1-specific shRNA (shENPP1) or the non-targeting control shRNA (shNC) were established using the pLKO.1-based lentiviral shRNA technique. The plasmid used to knockdown ENPP1 and the corresponding negative control plasmid were purchased from Sigma. The human *ENPP1* shRNA sequences were as follows: 5′-CGTGCATAGAACCAGAACATA-3′ (shENPP1-site1), 5′-TGAGGGACGATCTTTGAATAT-3′ (shENPP1-site2), 5′-CATGGGTTGAAGAATTGTTAA-3′ (shENPP1-site3), 5′-CTGCGAAAGTATGCTGAAGAA-3′ (shENPP1-site4) and 5′-GCTCATGTAACCCTTCGATTT-3′ (shENPP1-site5). The control shRNA sequence was as follows: 5′-GAAATGTACTGCGCGTGGAGAC-3′. Briefly, the recombinant lentiviruses were produced according to the manufacturer's instructions. In knockdown experiments, 293T cells were transfected with the expression constructs of shNC or shENPP1 and MCF7-luc and MCF7-luc anti-miR-27b-DR cells were infected with recombinant lentiviruses expressing shNC or shENPP1.

### Cell culture

MCF7, ZR75-1 and MDA-MB-231 cells were obtained from American Type Culture Collection, and 293T cells were obtained from Clontech. Each cell line was grown under the culture conditions recommended by the manufacturer. Metformin (1,1-dimethylbiguanide hydrochloride; Sigma-Aldrich) was used at 0.1–100 mM and docetaxel (Sanofi Aventis) was used at 1–10 nM.

### Real-time reverse transcription–PCR

Total RNA and miRNA were isolated from cells and tumour tissues using the RNeasy Mini Kit (QIAGEN), and cDNA was produced using the ExScript RT reagent Kit (Takara) or the TaqMan MicroRNA Reverse Transcript Kit (Applied Biosystems). TaqMan probes were obtained from Applied Biosystems. The cDNA samples were subjected to real-time PCR using SYBR Premix Ex Taq (Takara). The specific primers are listed in Supplementary Table 4. TaqMan MicroRNA Assays were used for qRT–PCR analyses of miRNAs (Applied Biosystems). Reactions were performed on the 7300 Real-Time PCR System (Applied Biosystems). Expression levels were normalized to those of *GAPDH* or *RNU6B*, and relative expression was calculated using the 2^ΔΔCt^ method.

### Mammosphere culture

MCF7 cells and their derivatives were seeded into 100-mm ultra-low attachment culture dishes (Corning) in Mammary Epithelial Cell Growth Medium (Lonza) at a density of at 2 × 10^4^ cells per ml.

### Co-immunoprecipitation

Extracts of 293T cells were prepared in immunoprecipitation buffer (20 mM Tris-HCl, pH 8.0, 150 mM NaCl, 1 mM EDTA, 0.1% NP-40, 10% glycerol, 1 mM DTT, protease inhibitor cocktail and phosphatase inhibitor). The supernatants were incubated with anti-HA or anti-Flag agarose (MBL) under rotation for 4 h at 4 °C. After washing four times with immunoprecipitation buffer, the immunoprecipitated protein complex bound to the beads was eluted with an HA or Flag peptide (MBL). The eluates and cell lysates were immunoblotted with anti-Flag, anti-HA, anti-ENPP1, anti-ABCG2 and anti-GFP antibodies.

### *In vitro* binding assay

ABCG2-HA was transcribed and translated using the TNT T7 Insect Cell Extract Protein Expression System (Promega). After affinity purification of ABCG2-HA from extracts of SF21 insect cells and ENPP1-MF or GFP-F from extracts of 293T cells, co-immunoprecipitation was performed as described above.

### Proximity ligation assay

The proximity ligation assay was performed using the Duolink kit (Sigma). MCF7-luc anti-NC cells and MCF7-luc anti-miR-27b cells were transfected with pCMV6-ABCG2-HA. At 48 h post-transfection, the cells were processed for immunostaining using anti-ENPP1 (1:250; sc-393419, Santa Cruz Biotechnology) and anti-HA (1:500; 3724, CST) primary antibodies. Incubation with the proximity ligation assay probes, hybridization, ligation and amplification were performed according to the manufacturer's protocol. Detection was performed using a fluorescence microscope (BZ-X700, Keyence).

### Dual-luciferase assay

MCF7 cells (3 × 10^3^) were seeded into a 96-well plate and co-transfected with 50 ng of the pTK-GLuc-27bs vector and 50 ng of a SV40 promoter-driven secreted *Cypridina* luciferase reporter vector (pSV40-CLuc; NEB) as an internal control using Lipofectamine 2000 reagent (Invitrogen). For the 3′UTR assay, MCF7 cells (3 × 10^3^) were seeded into a 96-well plate and co-transfected with pTK-GLuc-ENPP1-3′UTR, the miRNA expression vector, and pSV40-CLuc as an internal control using Lipofectamine 2000. Luciferase activity was quantified using a dual-luciferase assay system (NEB) and relative transactivation was calculated according to the manufacturer's instructions. All experiments were repeated at least three times.

### Fluorescence-activated cell sorting

An APC-conjugated anti-CD44 antibody (BD Bioscience, clone G44-26), a PE-Cy7-conjugated anti-CD24 antibody (BioLegend, clone LM5), an APC-conjugated anti-ABCG2 antibody (clone 5D3, BD Bioscience) and propidium iodide (5 μg ml^−1^; BD Bioscience) were used for fluorescence-activated cell sorting analyses. To detect the SP fraction, MCF7 and ZR75-1 cells were stained with 5 μg ml^−1^ Hoechst 33342 (Invitrogen) in the presence or absence of 1 μM Ko143 (Sigma) at 37 °C for 90 min. Flow cytometric analysis and cell sorting were performed using a JSAN cell sorter (Bay bioscience) and the results were analysed with FlowJo software.

### Microarray analyses

Total RNAs (50 ng) from MCF7-luc derivatives (MCF7-luc anti-NC, MCF7-luc anti-miR-27b, MCF7-luc miR-27b o.e. and MCF7-ENPP1-MF cells) were labelled and amplified using the Low Input Quick Amp labelling kit (Agilent Technologies). The Cy3-labelled RNAs were resuspended in 40 μl of hybridization solution (Agilent Technologies), applied to a SurePrint G3 Human GE 8 × 60 K array (Agilent Technologies) and covered with a Gasket 8-plex slide (Agilent Technologies). The slides were hybridized for 17 h at 65 °C, washed with Gene Expression Wash Buffer 1 (Agilent Technologies) for 1 min at room temperature, washed with Gene Expression Wash Buffer 2 (Agilent Technologies) for 1 min at 37 °C and then air-dried. The arrays from three independent experiments were analysed using microarray scanner (Agilent Technologies). Gene expression levels were calculated using Feature Extraction version 10.7.3.1 (Agilent Technologies). The normalized and log-transformed intensity values were then analysed using GeneSpring GX 7.3.1 (Agilent Technologies).

### Single-cell PCR

After single-cell sorting using a JSAN cell sorter, qRT–PCR was performed as described previously^60^.

### Bioluminescent imaging

All animal experiments were performed in accordance with the guidelines of the Institute for Laboratory Animal Research, National Cancer Center Research Institute. Female NOD/SCID mice (NOD.CB17-Prdkc^scid^/J; CLEA Japan) aged 4–6 weeks were used for the xenograft model. Images were analysed with Living Image software (Caliper Life Sciences).

### Mammary fat pad xenografts

MCF7 cells and their derivatives were suspended in a 1:1 mixture of PBS/Matrigel (Sigma) and injected into the mammary fat pads of NOD/SCID mice (*n*=4 animals; 50 μl and 10^5^ cells per animal). All animal work followed a protocol approved by the National Cancer Center Institutional Animal Care and Use Committee (T11-024, T11-024-C01 and T14-001).

### Patients and tissue samples

Twenty-six patients who underwent primary breast surgery for stage II–III invasive breast carcinoma between 1996 and 2000 in the National Cancer Center Hospital in Japan were selected. The study was approved by the Institutional Review Board of the National Cancer Center (No. 2010-128) and informed consent was obtained from all patients. To evaluate miR-27b expression in MCF7 cells, RNA samples from normal breast tissues were purchased from Wako and Agilent. For the qRT-PCR validation study, cDNAs from 53 human primary breast tumours were purchased from OriGene. To investigate the expression and localization of ENPP1 in breast cancer patient tissues, a tissue microarray was purchased from SUPER BIO CHIPS. For survival analyses, relapse-free survivals in luminal A, luminal B and basal-type cancer patients (grade 3) were stratified by expression of the gene of interest and presented as Kaplan–Meier plots^47^.

### Statistical analysis

Data are presented as the mean±s.d. Unless otherwise stated, statistical significance was determined by a Student's two-tailed *t*-test. *P*<0.05 was considered statistically significant.

## Additional information

**Accession codes:** Microarray data have been deposited in the Gene Expression Omnibus (GEO) database under accession code GSE67631.

**How to cite this article:** Takahashi, R.-u. *et al*. Loss of microRNA-27b contributes to breast cancer stem cell generation by activating ENPP1. *Nat. Commun.* 6:7318 doi: 10.1038/ncomms8318 (2015).

## Supplementary Material

Supplementary Information Supplementary Figures 1-15 and Supplementary Tables 1-4
