# Supplementary material for: Loss of microRNA-27b contributes to breast cancer stem cell generation by activating ENPP1
Source: Nat Commun. 2015 Jun 12;6:7318. doi: 10.1038/ncomms8318 (PMC4490376; doi:10.1038/ncomms8318)
Supplement: Supplementary Information — Supplementary Figures 1-15 and Supplementary Tables 1-4 [file ncomms8318-s1.pdf]

Supplementary Fig. 1

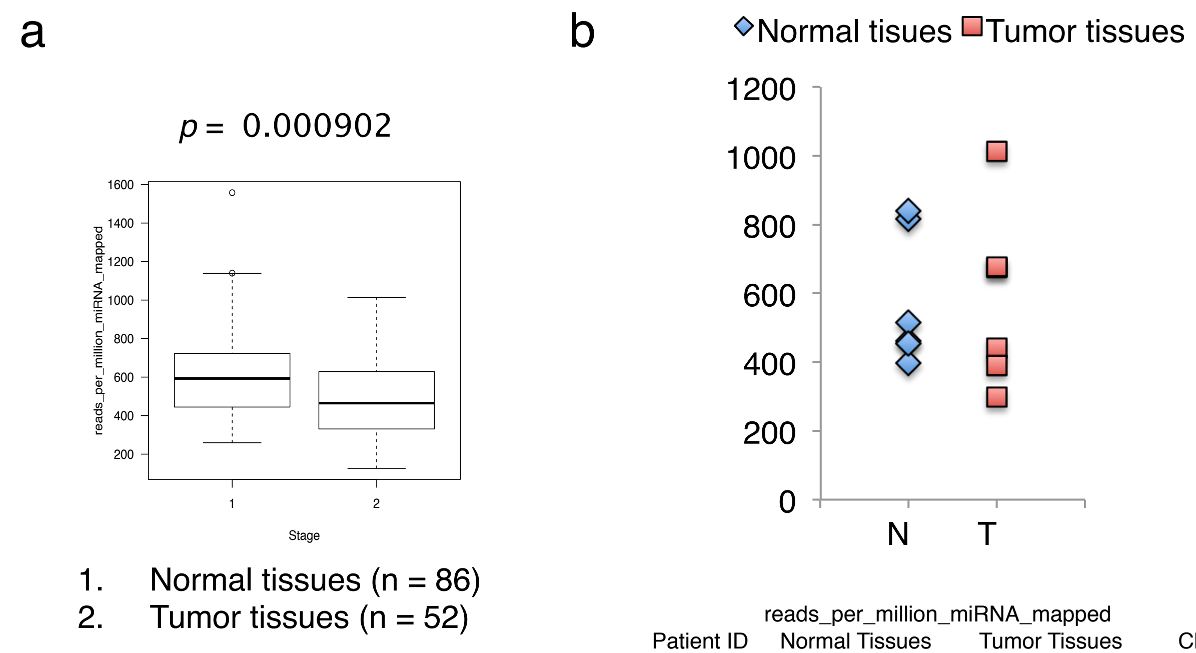

	reads_per_million_miRNA_mapped		
Patient ID	Normal Tissues	Tumor Tissues	Chage
1	460.51	1014.00	Up
2	454.43	676.38	Up
3	818.19	440.36	Down
4	513.51	389.63	Down
5	398.91	299.37	Down
6	840.14	673.87	Down

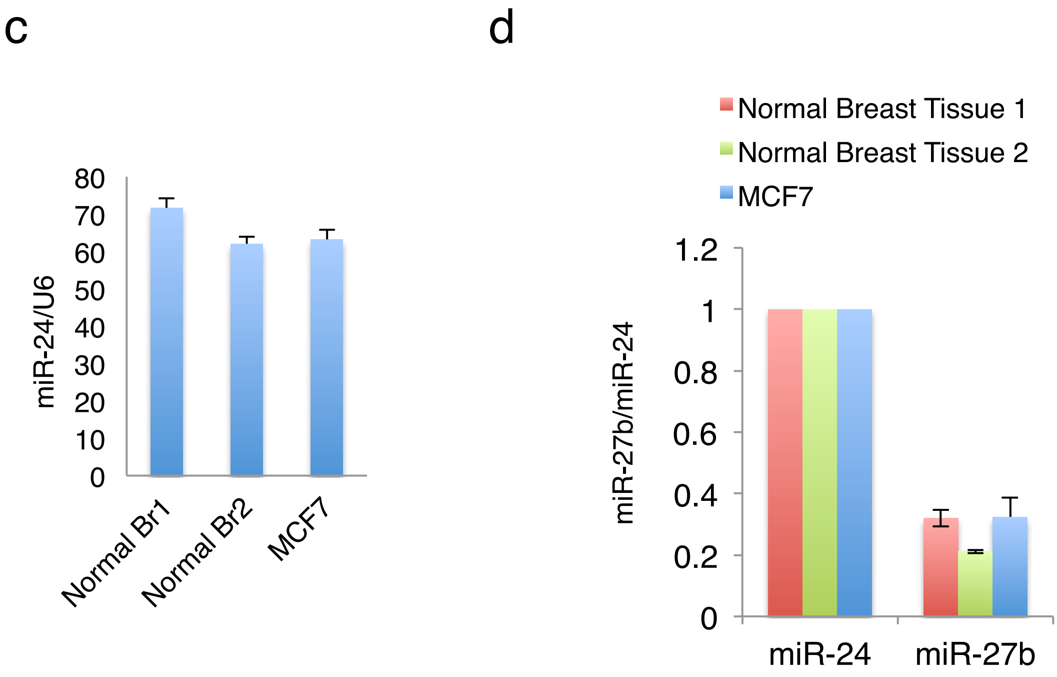

Supplementary Fig. 1. Expression profile of miR-27b in breast cancer patients.
(a) Data from The Cancer Genome Atlas showing the miR-27b expression in luminal type breast cancer patients (n = 52) who received the taxane-based adjuvant chemotherapy. The *P*-value was calculated using Mann-Whitney *U* test.
(b) The expression level of miR-27b among the six matched pair cases that contained normal and cancer tissues in (a).
(c, d) Expression profiles of miR-24 and miR-27b in normal breast tissues and MCF7 cells. Data are represented as the mean ± SD of n = 3 replicates.

Supplementary Fig. 2

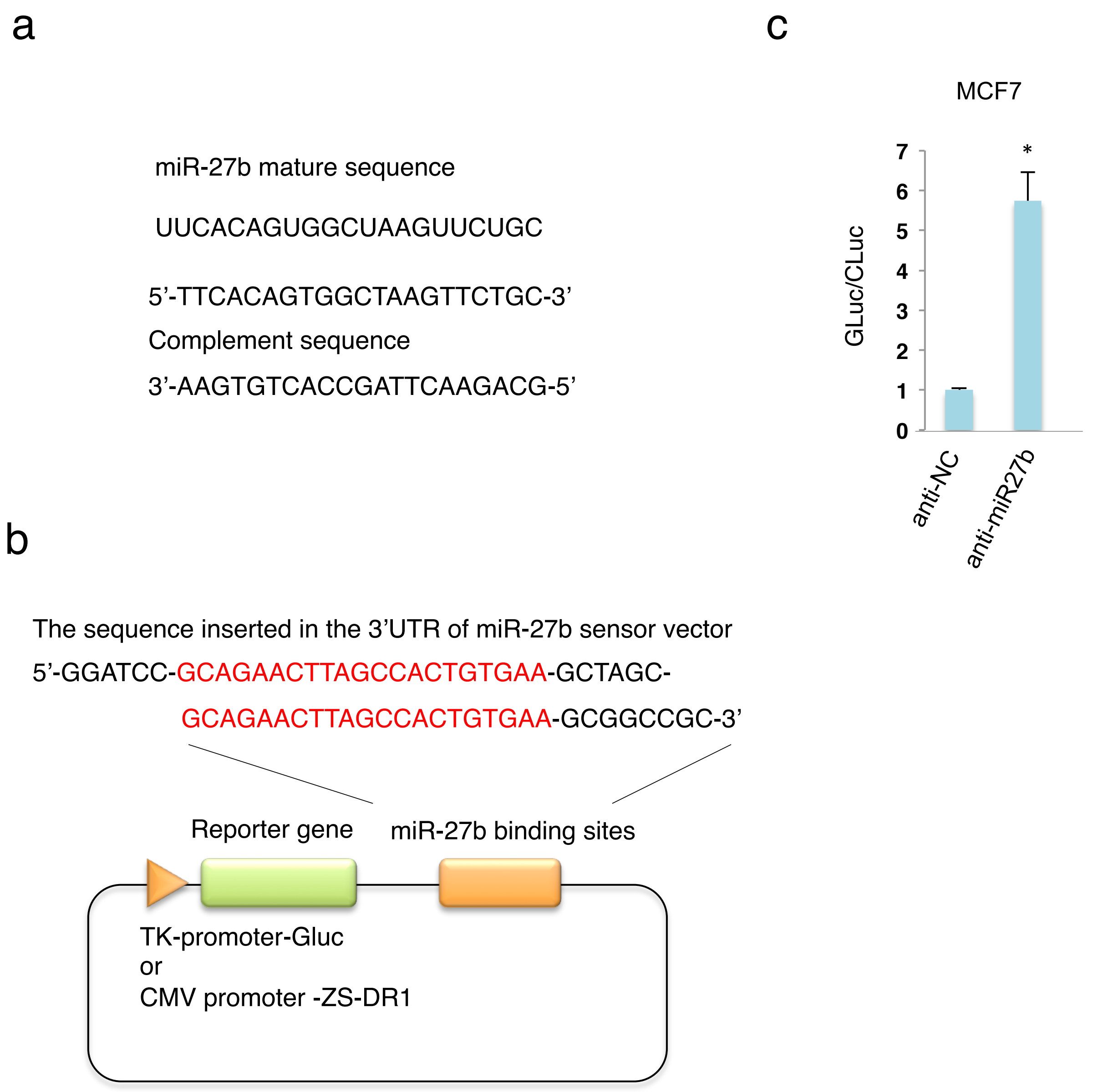

Supplementary Fig. 2. Establishment of miR-27b knockdown cell lines.
(a, b) A schematic illustration of the miR-27b sensor construct used in the experiment shown in (b).
(c) A dual-luciferase assay showing the efficiency of knockdown of miR-27b in MCF7-luc anti-miR-27b cells. Control cells (MCF7-luc anti-NC) stably expressed a non-specific antisense sequence. The cells were co-transfected with the pTK-GLuc-27bs and pSV40-CLuc vectors expressing *Gaussia* luciferase (GLuc) and *Cypridina* luciferase (CLuc), respectively. The level of miR-27b expression was determined as the ratio of GLuc to CLuc activity. Data are represented as the mean ± SD of n = 3 replicates. Statistical significance was determined by Student's *t*-test (* $P < 0.05$).

Supplementary Fig. 3

a

miR-27b mature sequence

UUCACAGUGGCUAAGUUCUGC

PPARG 3’UTR

NM_138711.3

5’_CAGAGAGTCCTGAGCCACTGCCAACATTTCCCTTC

TTCCAGTTGCACTATTCTGAGGGAAAATCTGACACCT

miR-27b target site

AAGAAATTT**ACTGTGAA**AAAGCATTTTAAAAAGAAAA

GGTTTTAGAATATGATCTATTTTATGCATATTGTTTATAA

AGACACATTTACAATTTACTTTTAATATTAAAAATTACCA

TATTATGAAATTGCTGATAGTA_3’

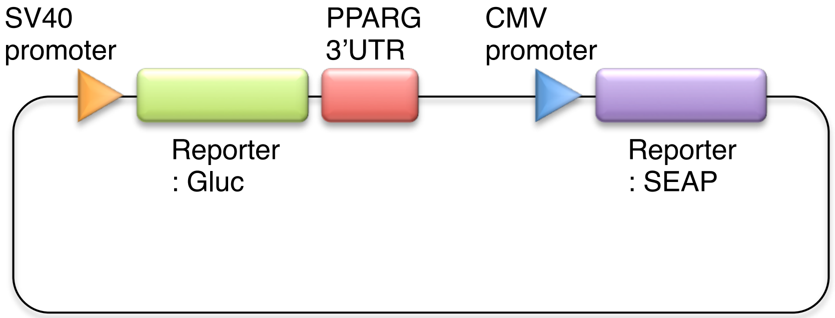

b

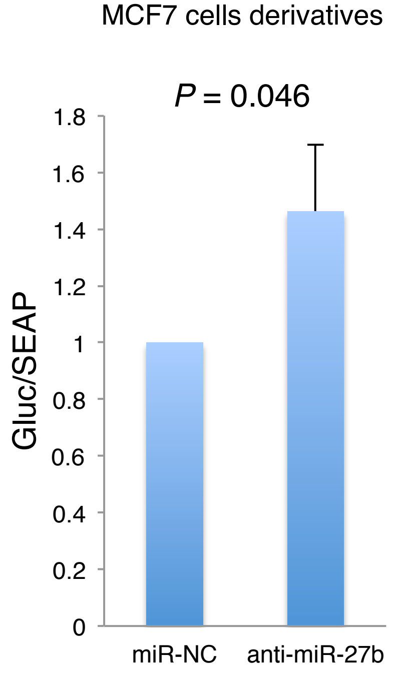

c

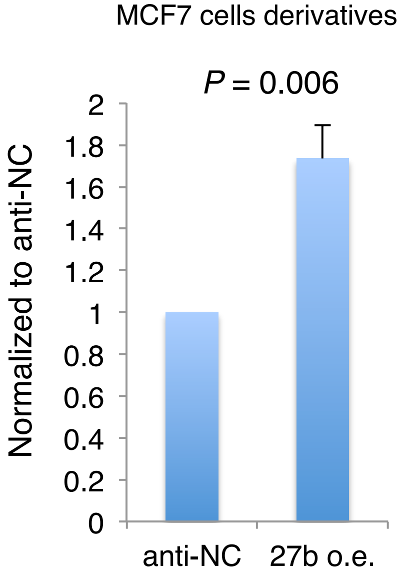

d

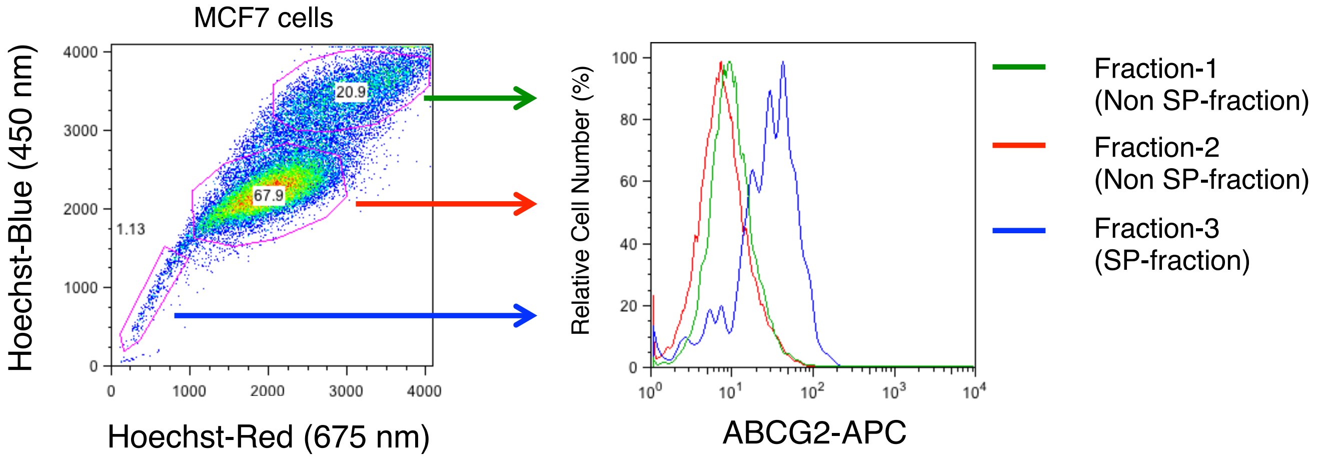

Supplementary Fig. 3. Construction of the *PPARG* 3'UTR vector.

(a) A schematic illustration of the *PPARG* 3'UTR vector.

(b) A dual-luciferase assay showing the efficiency of knockdown of miR-27b in MCF7-luc anti-miR-27b cells. MCF7-luc anti-NC or MCF7-luc anti-miR-27b cells were transfected with the *PPARG* 3'UTR vector shown in (a). The level of miR-27b expression was determined as the ratio of *Gaussia* luciferase (GLuc) to *Cypridina* luciferase (CLuc) activity. Data are represented as the mean ± SD of n = 3 replicates. Statistical significance was determined by Student's *t*-test.

(c) Expression of miR-27b in MCF7-luc miR-27b o.e. cells. Data are represented as the mean ± SD of n = 3 replicates. Statistical significance was determined by Student's *t*-test.

(d) Flow cytometric analysis of ABCG2 expression in the SP-fraction of MCF7 cells. FSC, forward scatter.

Supplementary Fig. 4

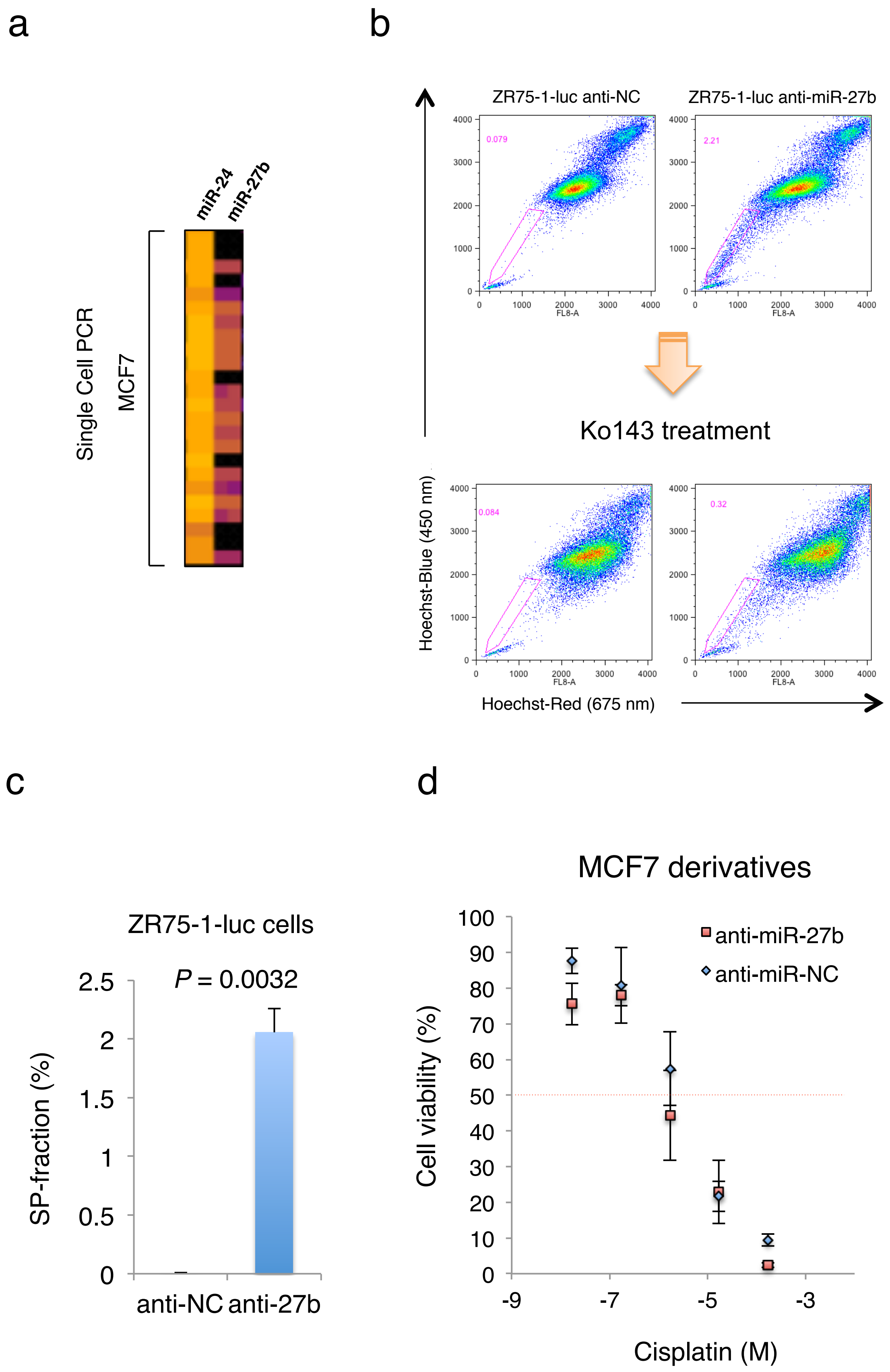

Supplementary Fig. 4. Down-regulation of miR-27b enhances the SP-fraction of ZR75-1 cells.
(a) Single cell qRT-PCR analyses of miR-24 and miR-27b expression in MCF7 cells. Expression levels were normalized to those of *RNU6B*.
(b) Flow cytometric analysis of the SP-fractions of ZR75-1-luc derivatives.
(c) Quantification of the SP-fractions of ZR75-1-luc derivatives. The SP-fraction was determined as the difference between the level of Hoechst 33342 staining in the presence and absence of the ABCG2 inhibitor Ko143. Data are represented as the mean ± SD of n = 3 replicates. Statistical significance was determined by Student's *t*-test.
(d) Dose-response curves of MCF7-luc anti-NC and MCF7-luc anti-miR-27b cells treated with cisplatin. Cell viability was normalized to that of the corresponding cells treated with DMSO. The red dashed line indicates the IC_{50} value. Data are represented as the mean ± SD of n = 3 replicates.

Supplementary Fig. 5

a

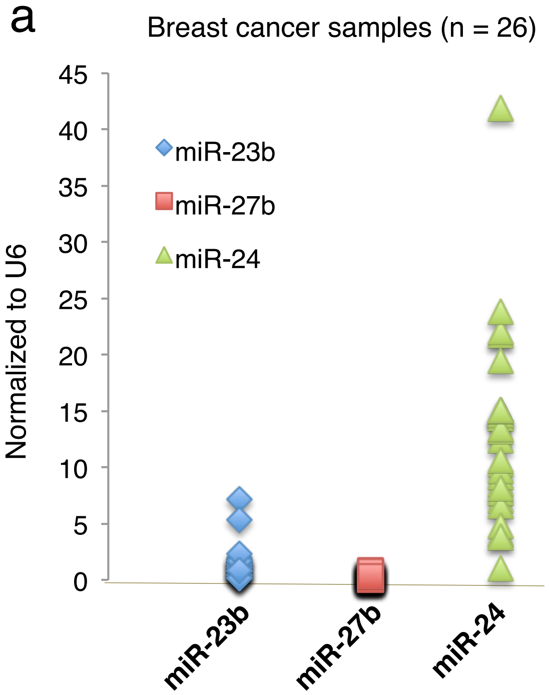

b

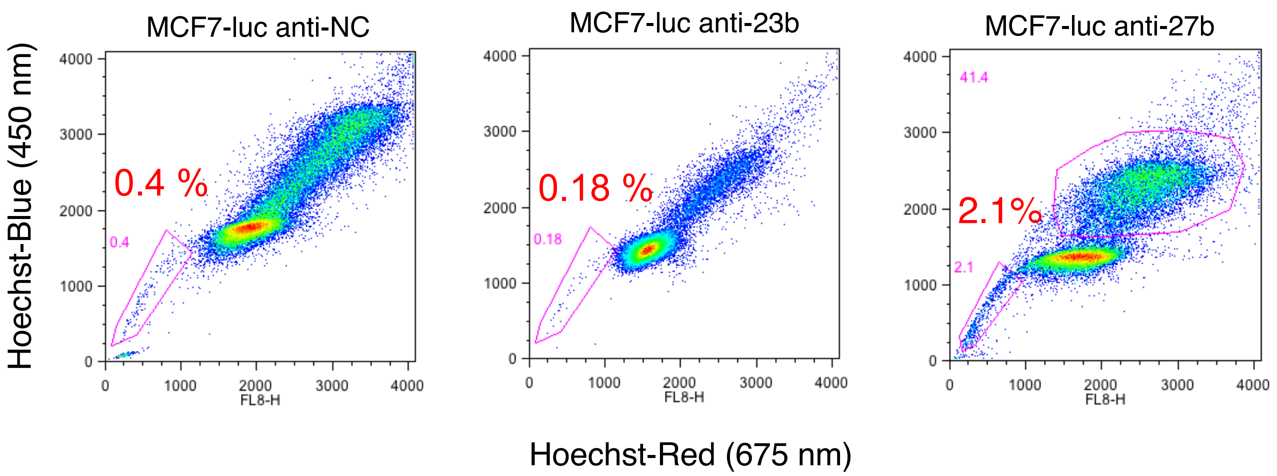

Supplementary Fig. 5. Functional analysis of miR-23b in MCF7 cells.

(a) Expression levels of miR-23b, miR-27b and miR-24 in NCC patients (n = 26).

(b) Flow cytometric analyses of the SP-fractions of MCF7-luc derivatives.

Supplementary Fig. 6

a

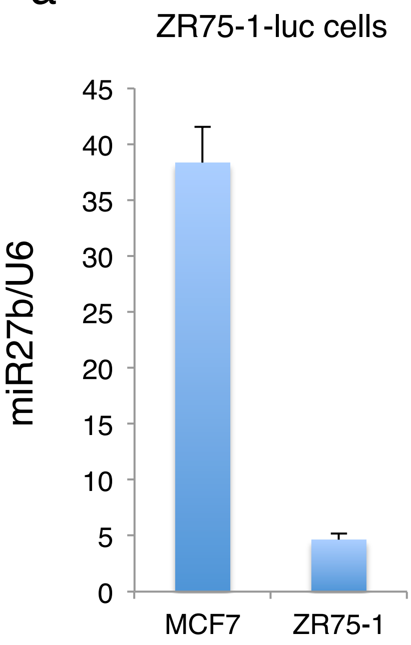

b

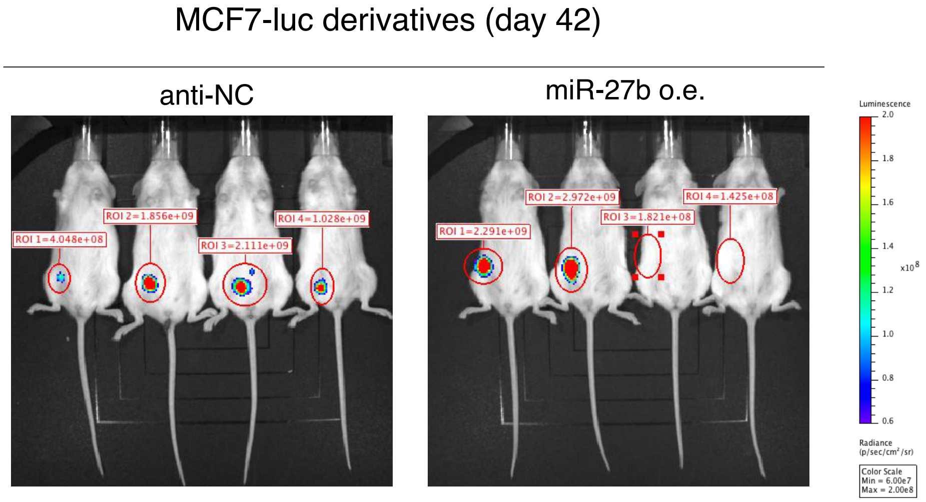

c

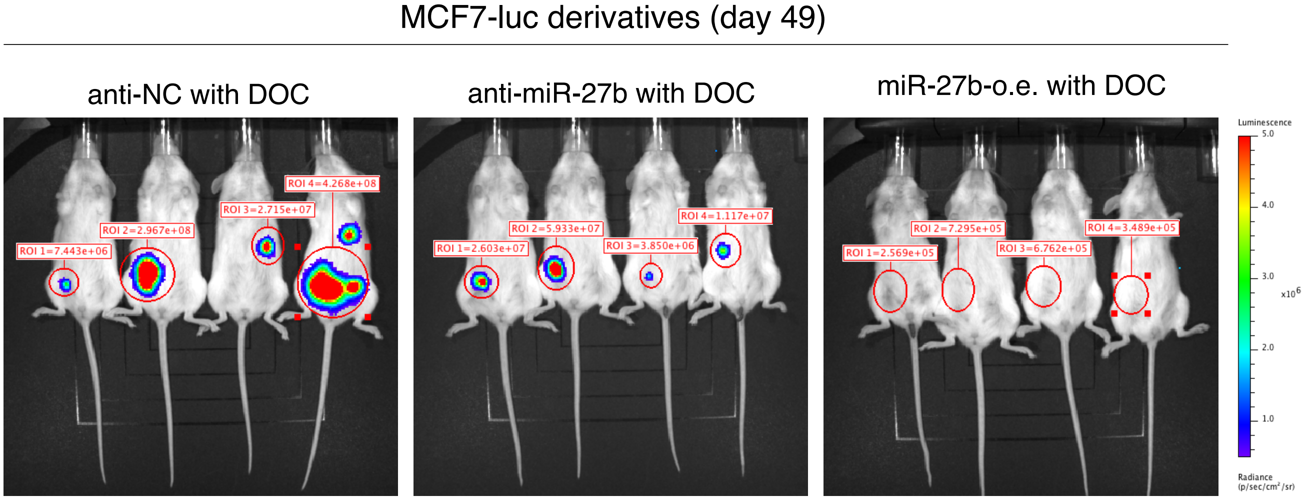

d

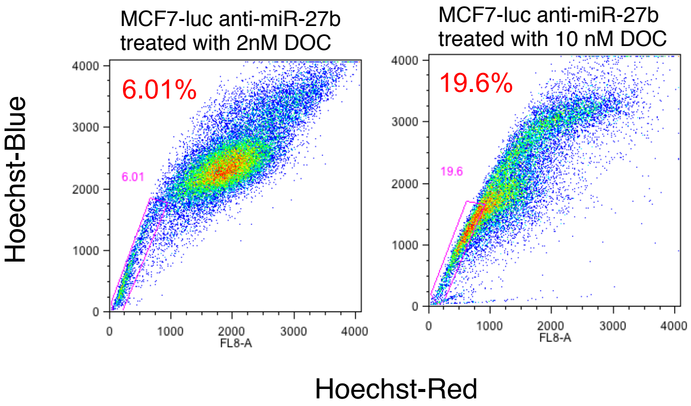

Supplementary Fig. 6. MiR-27b inhibits the generation of tumorigenic cells in the SP-fraction.
(a) A qRT-PCR analysis of relative miR-27b expression levels in MCF7 and ZR75-1 cells. The expression level of *RNU6B* was used for normalization. Data are represented as the mean ± SD of n = 3 replicates.
(b) Bioluminescent images of tumours in NOD/SCID mice injected with MCF7-luc cell derivatives. The cells were injected into the mammary fat pad of the mice (n = 4 animals and 10^5 cells per animal).
(c) Bioluminescent images of tumours in NOD/SCID mice injected with docetaxel-treated MCF7-luc cell derivatives. After exposure to 5 nM docetaxel for 4 days, the cells were injected into the mammary fat pad of the mice (n = 4 animals and 10^5 cells per animal).
(d) Flow cytometric analysis of the SP-fraction of MCF7-luc anti-miR-27b cells that were treated with 2 nM or 10 nM docetaxel for 96 h.

Supplementary Fig. 7

a

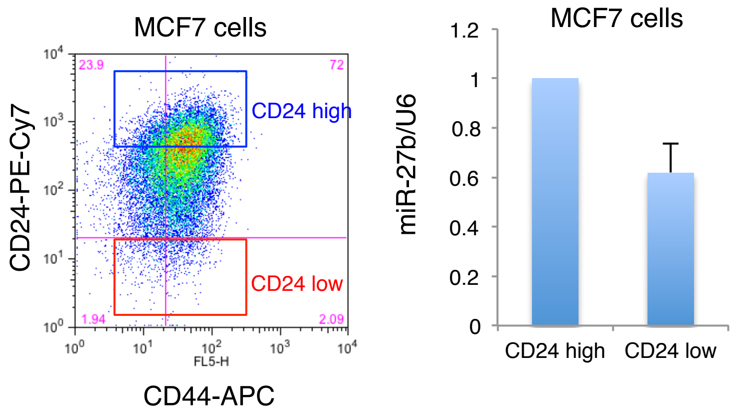

b

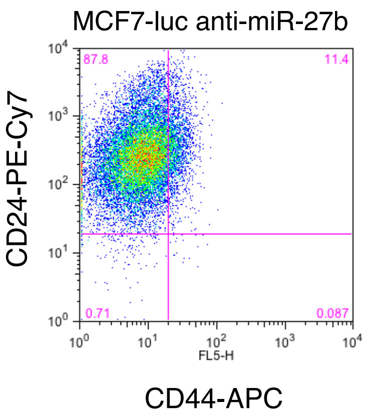

Supplementary Fig. 7. Expression level of miR-27b in CD24^{low} cell fraction.

(a) QRT-PCR analysis coupled with flow cytometry analysis in MCF7 cells.

(b) Flow cytometry analysis of CD44 and CD24 expression in MCF7-luc anti-miR-27b cells.

Supplementary Fig. 8

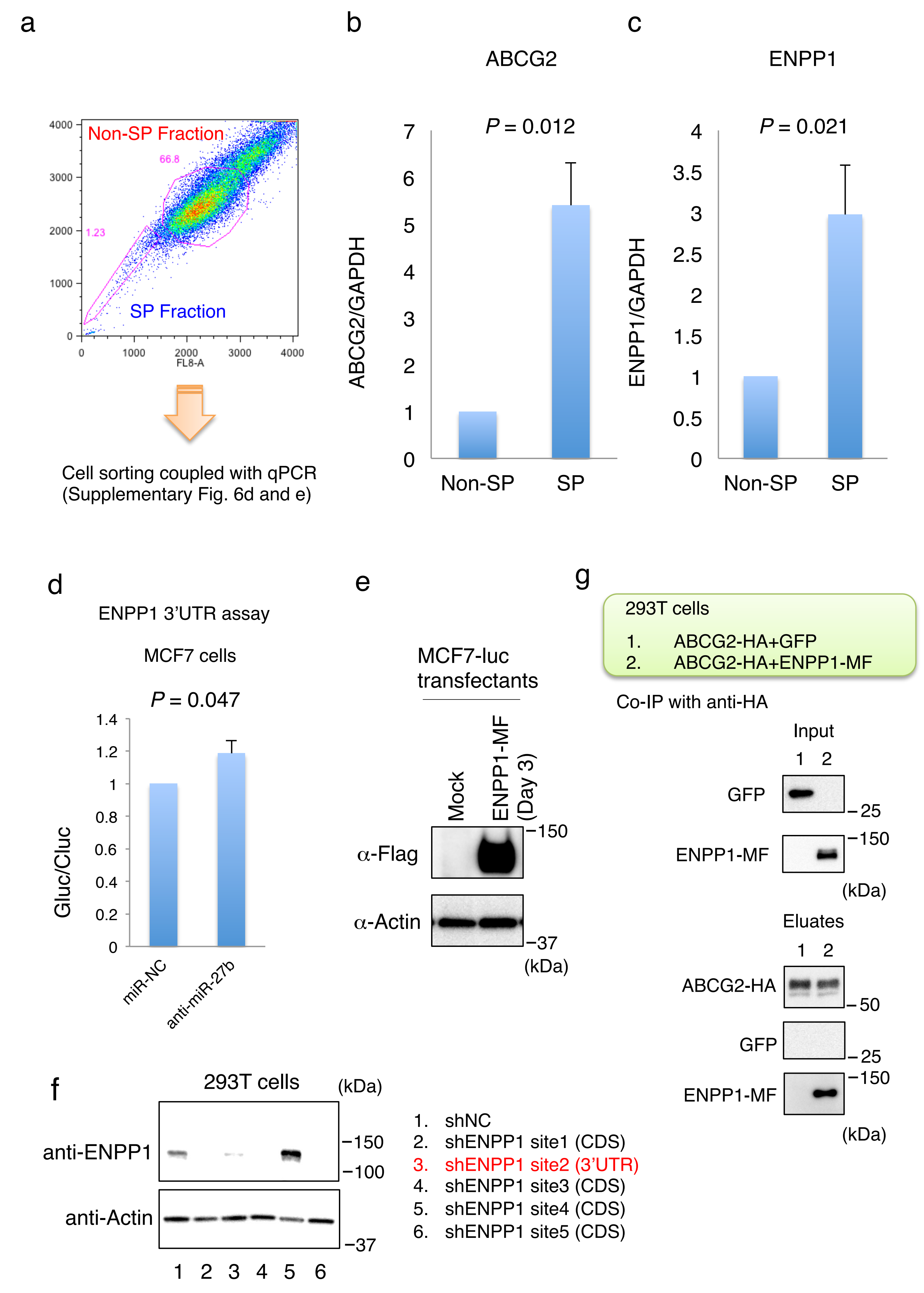

Supplementary Fig. 8. Functional analysis of ENPP1.

(a–c) The expression levels of *ENPP1* and *ABCG2* in the SP-fraction of MCF7 cells, as determined by qRT-PCR analysis coupled with cell sorting. Data are represented as the mean ± SD of n = 3 replicates. Statistical significance was determined by Student's *t*-test.

(d) Luciferase activities in MCF7 cells transfected with the pTK-GLuc reporter construct containing the wild-type 3'UTR of *ENPP1* (50 ng), an expression vector harbouring a miR-27b antisense sequence or a non-specific miRNA sequence (miR-NC), and the pSV40-cLuc vector (50 ng). The ratio of *Gaussia* to *Cypridina* luciferase activity was determined. Data are represented as the mean ± SD of n = 3 replicates. Statistical significance was determined by Student's *t*-test.

(e) Immunoblot analysis of ENPP1 expression in mock transfected MCF7-luc cells or MCF7-luc cells transiently expressing ENPP1-MF.

(f) Efficiency of knockdown of ENPP1 in 293T cells.

(g) Co-immunoprecipitation analyses of 293T cells expressing ENPP1-MF and ABCG2-HA.

Supplementary Fig. 9

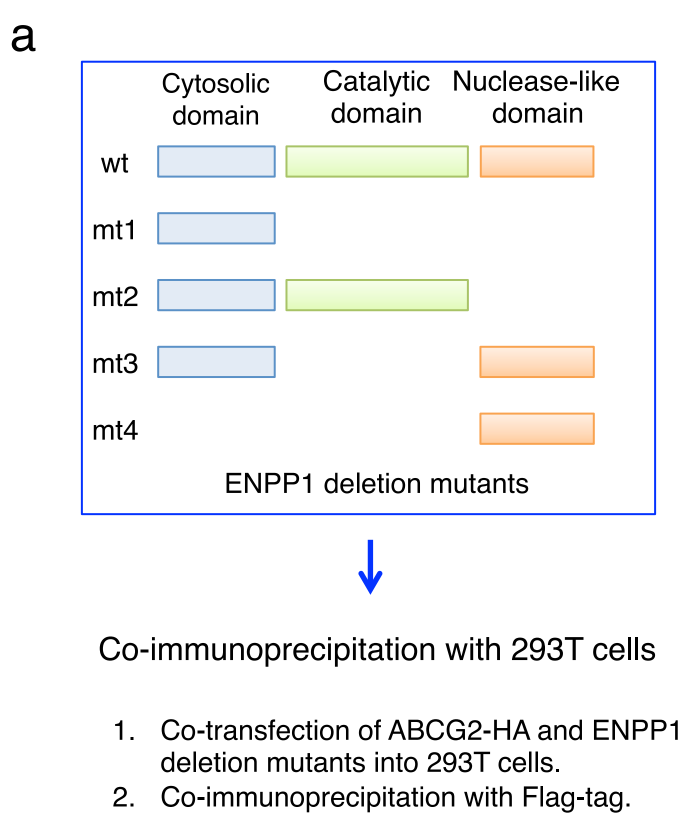

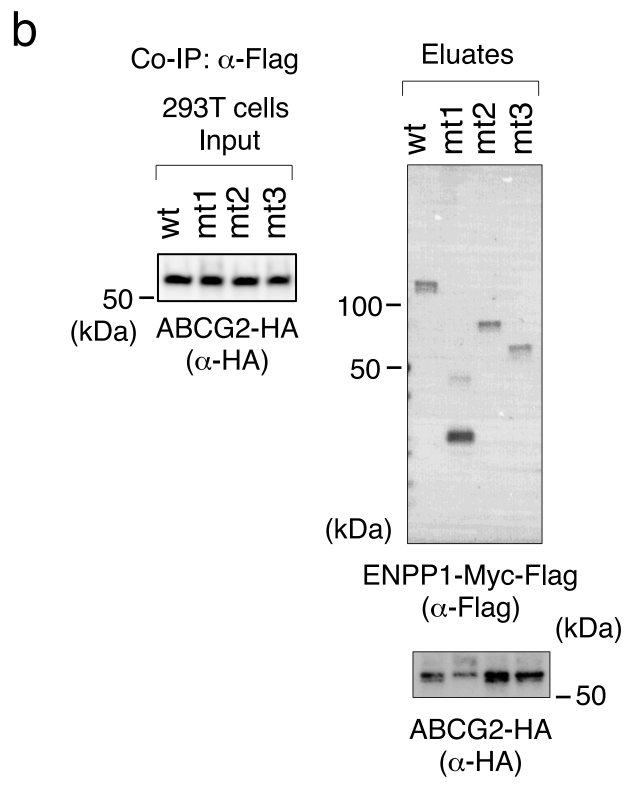

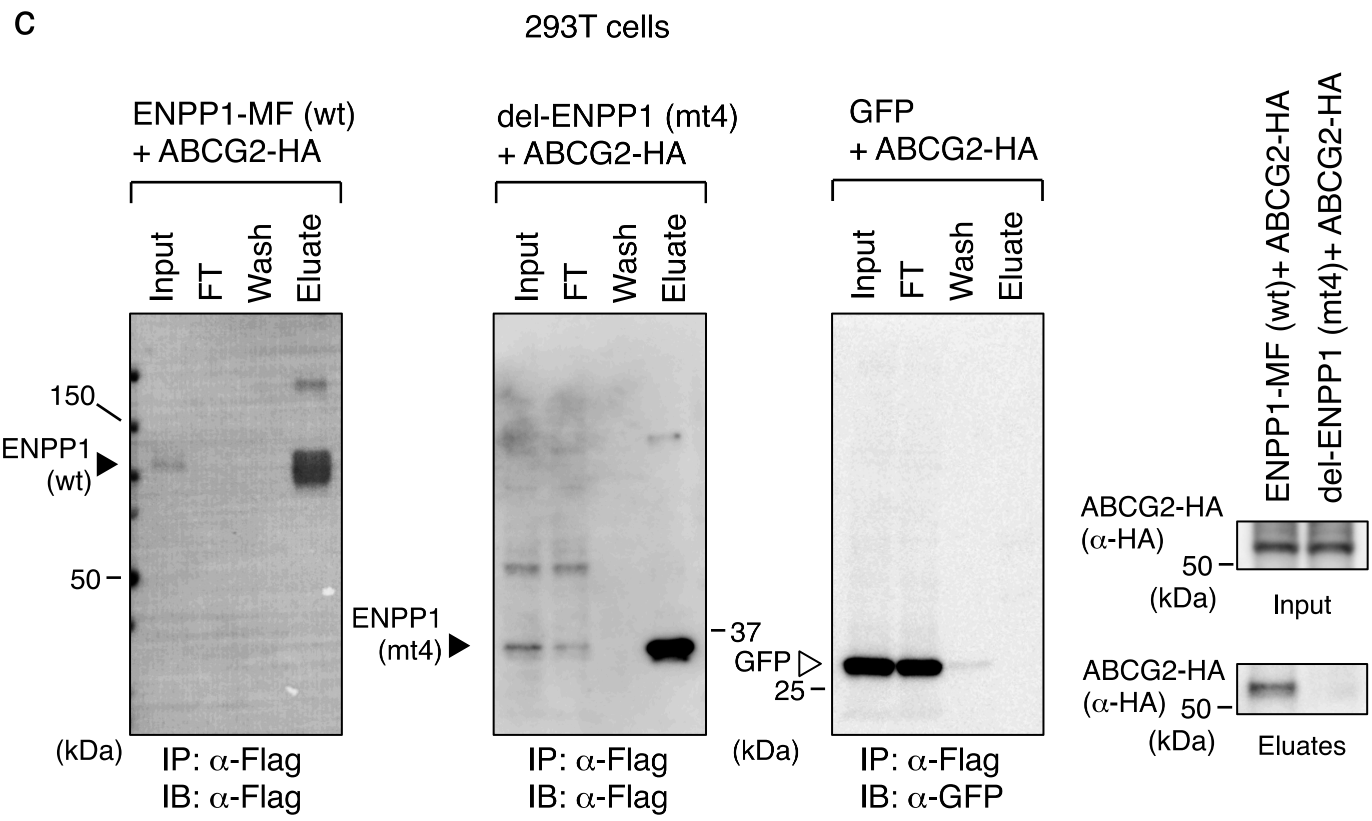

Supplementary Fig. 9. The N-terminal region of ENPP1 is important for the physical interaction with ABCG2.

(a) A schematic illustration of the ENPP1 deletion mutants used in the experiment shown in (b).

(b) Co-immunoprecipitation analyses of 293T cells expressing ABCG2-HA and the ENPP1 deletion mutants.

(c) Co-immunoprecipitation analyses of 293T cells expressing ABCG2-HA and ENPP1-MF, an ENPP1 deletion mutant (mt4), or GFP as a control.

Supplementary Fig. 10

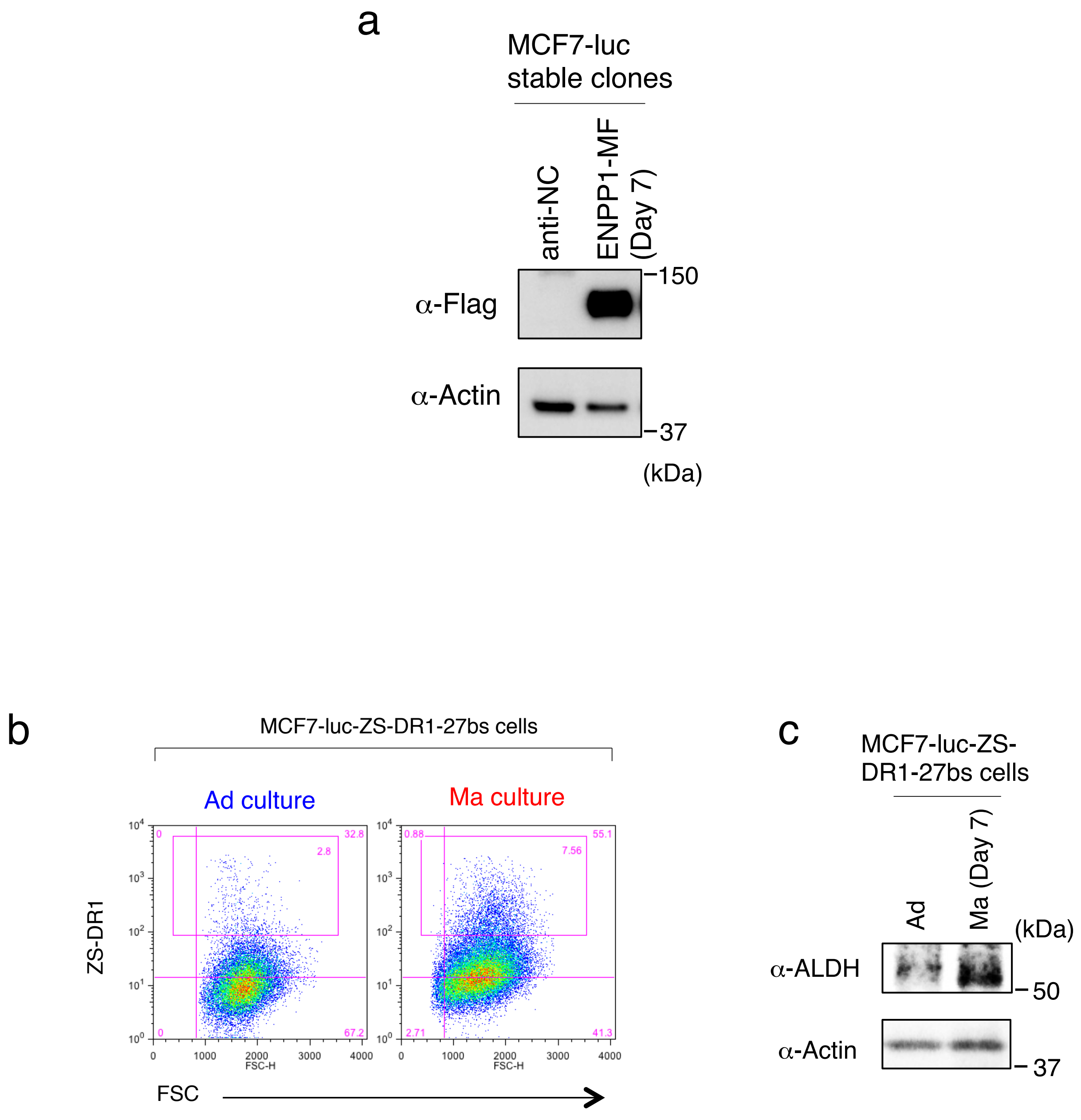

Supplementary Fig. 10. Mammosphere culture for CSC generation.

(a) Immunoblot analyses of ENPP1 expression in MCF7-luc cells stably expressing ENPP1-MF.

(b) Flow cytometric analysis of ZS-DR1 positive cells, representing those in which miR-27b was down-regulated, grown under adherent (Ad) and mammosphere (Ma) culture conditions.

(c) Immunoblot analysis of ALDH expression in MCF7-luc ZS-DR1-27bs cells grown under Ad or Ma culture conditions for 7 days. b-actin was used as a loading control.

Supplementary Fig. 11

a

MCF7-luc ENPP1-MF (day 34)

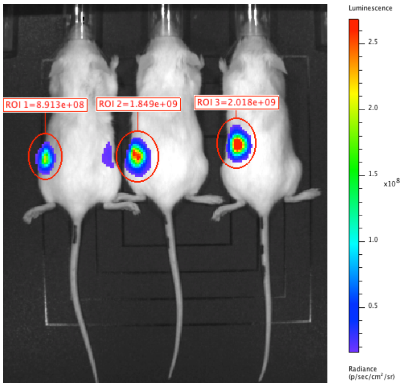

b

MCF7-luc derivatives (day 31)

MCF7-luc ZS-DR1-27bs (as technical control)

MCF7-luc shENPP1+DMSO

MCF7-luc shENPP1+DOC

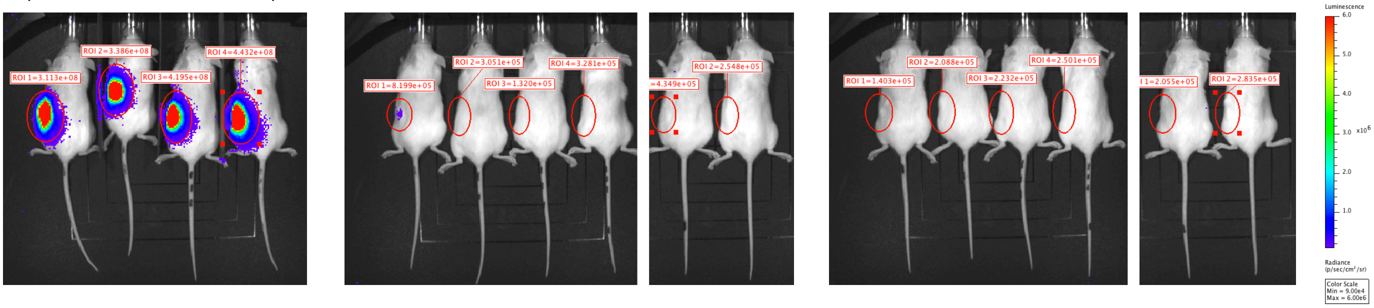

Supplementary Fig. 11. The role of ENPP1 in tumourigenicity of MCF7-luc cells.

(a) Bioluminescent images of tumours in NOD/SCID mice that were subcutaneously injected with MCF7-luc ENPP1-MF cells (n = 3 animals and 10^6 cells per animal).

(b) Bioluminescence images of tumours in NOD/SCID mice that were injected with or without docetaxel-treated MCF7-luc cell derivatives. After exposure to 5 nM docetaxel for 4 days, the cells were injected into the mammary fat pad of the mice (n = 6 animals and 10^5 cells per animal). MCF7-luc ZS-DR-27bs cells were used as a positive control (n = 4 animals and 10^5 cells per animal).

Supplementary Fig. 12

a

Figure 8b

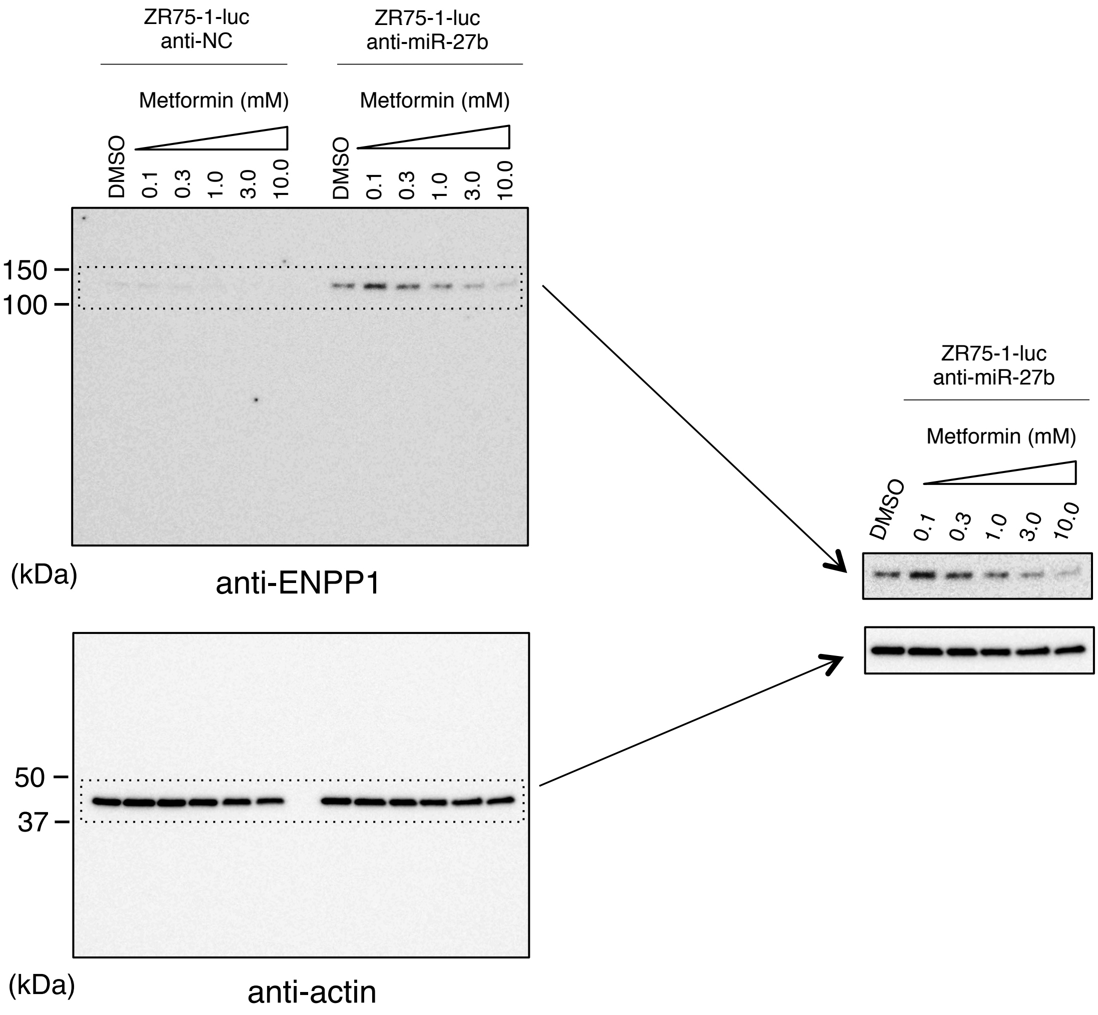

b

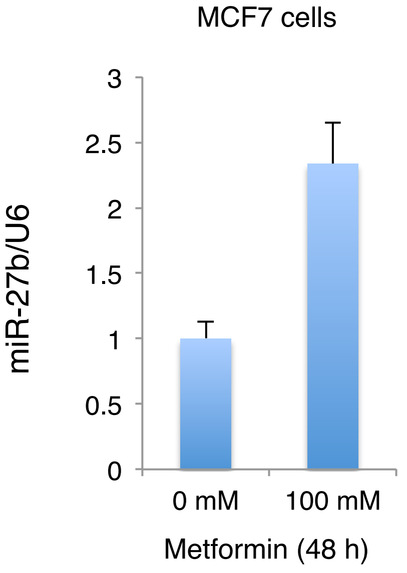

c

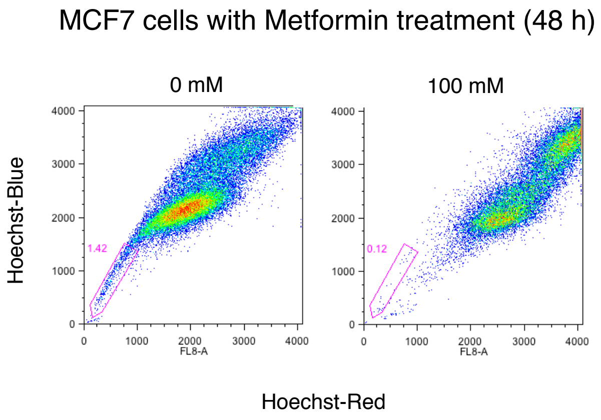

Supplementary Fig. 12. Metformin induces miR-27b-mediated suppression of ENPP1.

(a) Original data of western blot analysis in Figure 8b. Immunoblot analyses of ENPP1 expression in ZR75-1-luc anti-NC and anti-miR-27b cells incubated with metformin (0.1–10 mM) for 72 h. b-actin was used as a loading control.

(b) QRT-PCR analysis in MCF7 cells after 48 h incubation of 100 mM metformin. Data are represented as the mean ± SD of n = 3 replicates.

(c) Flow cytometric analysis of the SP-fractions of MCF7 cells after 48 h incubation of 100 mM metformin.

Supplementary Fig. 13

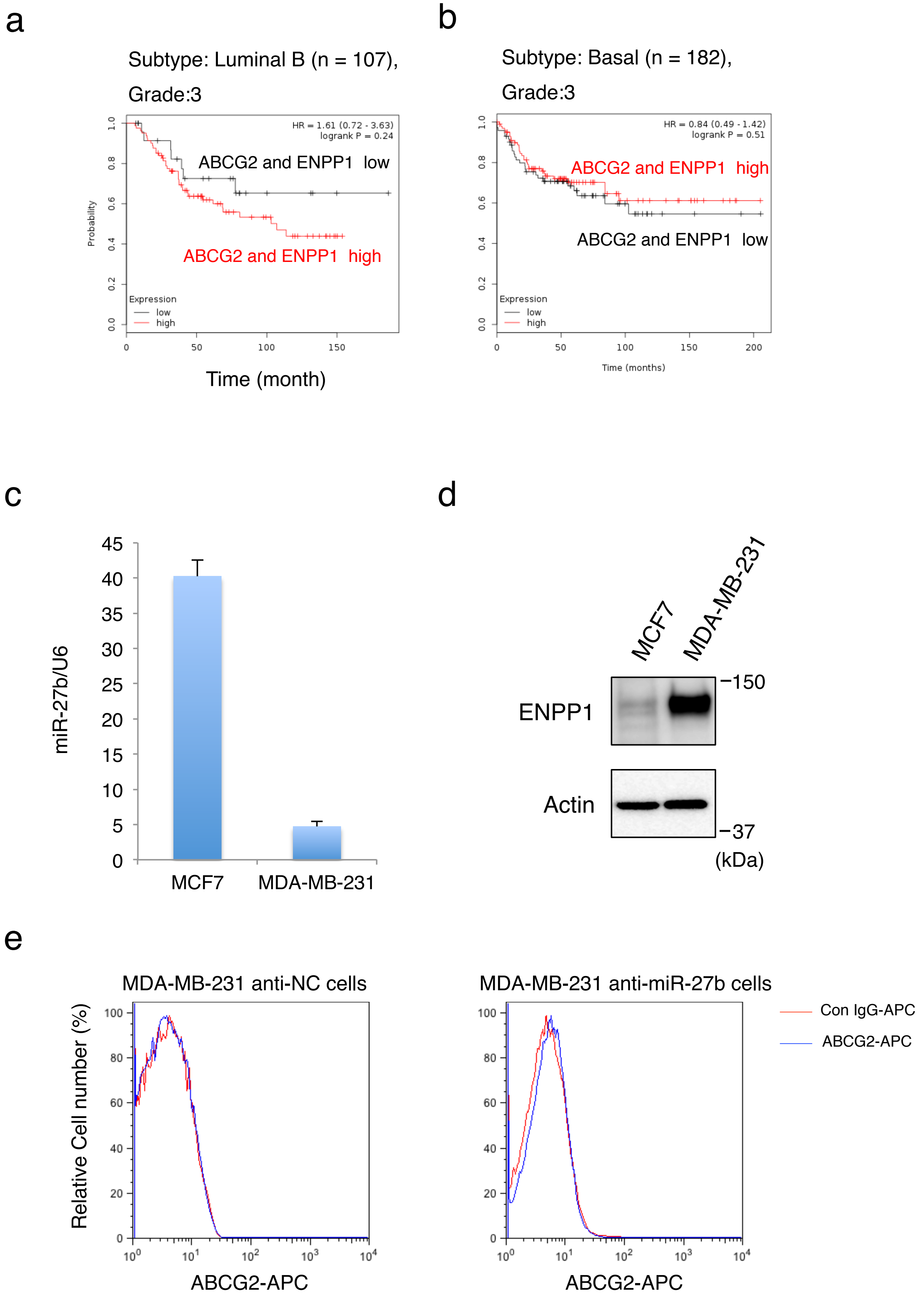

Supplementary Fig. 13. Evaluation of miR-27b, ENPP1 and ABCG2 expression in luminal B and basal-type breast cancer.

(a) Kaplan-Meier representation of the probability of recurrence-free survival in 107 luminal B-type breast cancer cases classified according to the expression levels of *ABCG2* and *ENPP1*. The *P*-value was calculated using a log rank test.

(b) Kaplan-Meier representation of the probability of recurrence-free survival in 182 basal-type breast cancer cases classified according to the expression levels of *ABCG2* and *ENPP1*. The *P*-value was calculated using a log rank test.

(c) A qRT-PCR analysis of relative miR-27b expression levels in MCF7 and basal-type MDA-MB-231 breast cancer cells. The expression level of *RNU6B* was used for normalization. Data are represented as the mean ± SD of n = 3 replicates.

(d) The expression level of ENPP1 protein in MCF7 and MDA-MB-231 cells.

(e) Flow cytometric analysis of MDA-MB-231 derivatives.

Supplementary Fig. 14

Patient ID	Diagnosis	Stage
Patient_1	Infiltrating ductal carcinoma	IIB
Patient_2	Infiltrating ductal carcinoma	IIA
Patient_3	Infiltrating ductal carcinoma	IIIC

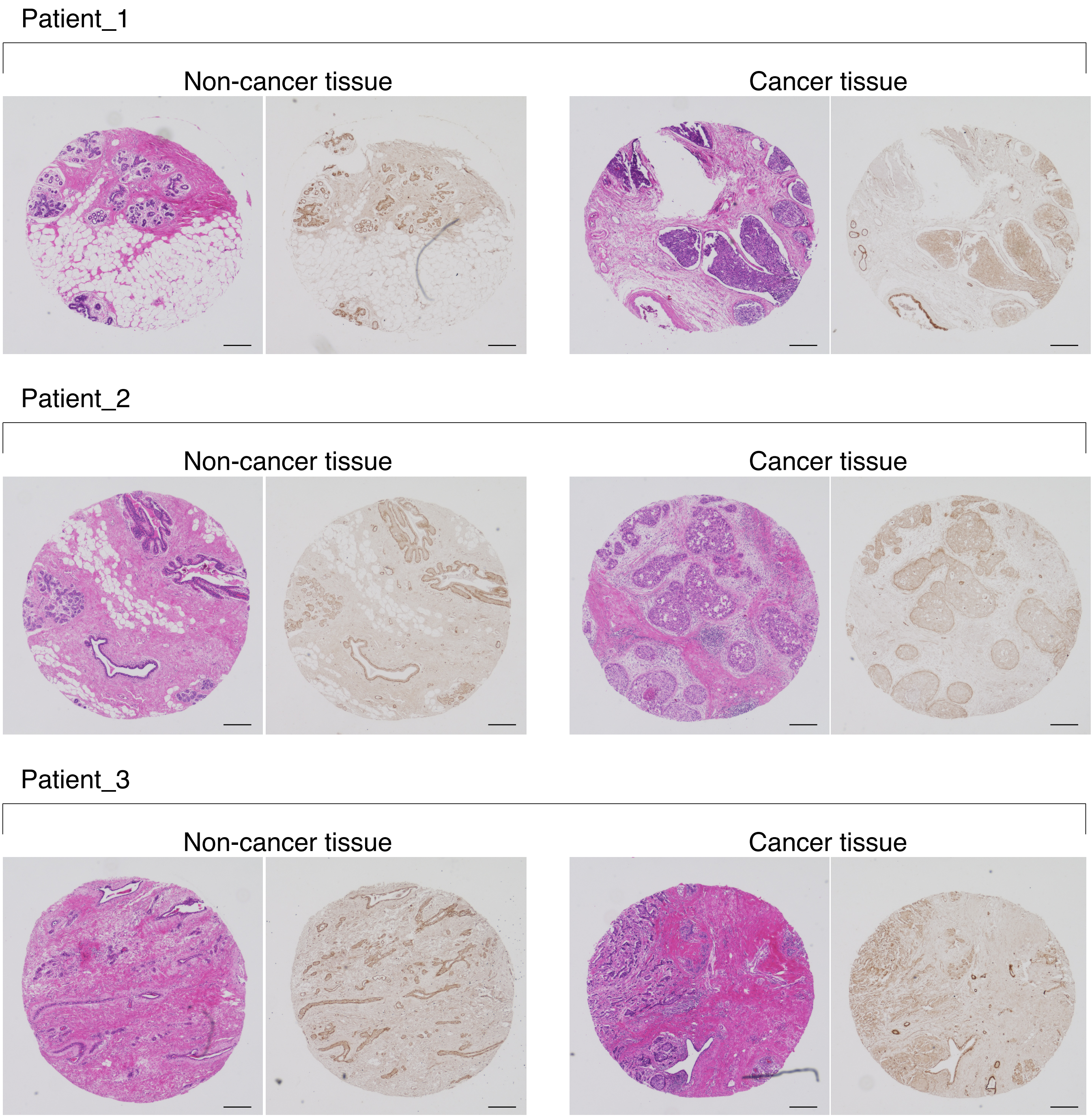

Supplementary Fig. 14. The expression of ENPP1 in breast cancer tissues.
Immunohistochemical staining of ENPP1 (brown) in normal breast and cancer tissues classified by the T-stage, as shown in the upper panel. Scale bar, 200 mm.

Supplementary Fig. 15

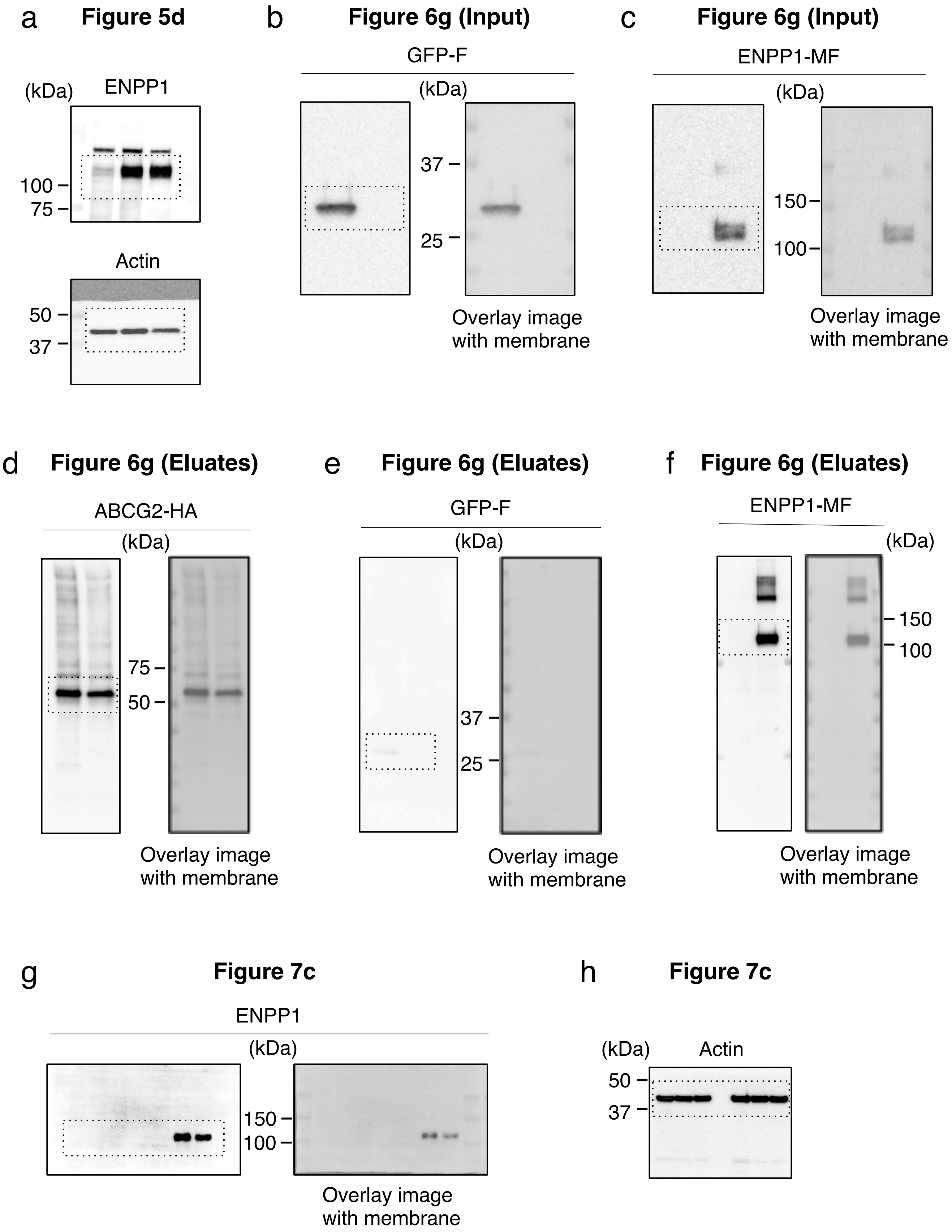

Supplementary Fig. 15. Original data of western blot analysis.

Supplementary Fig. 15 (continued)

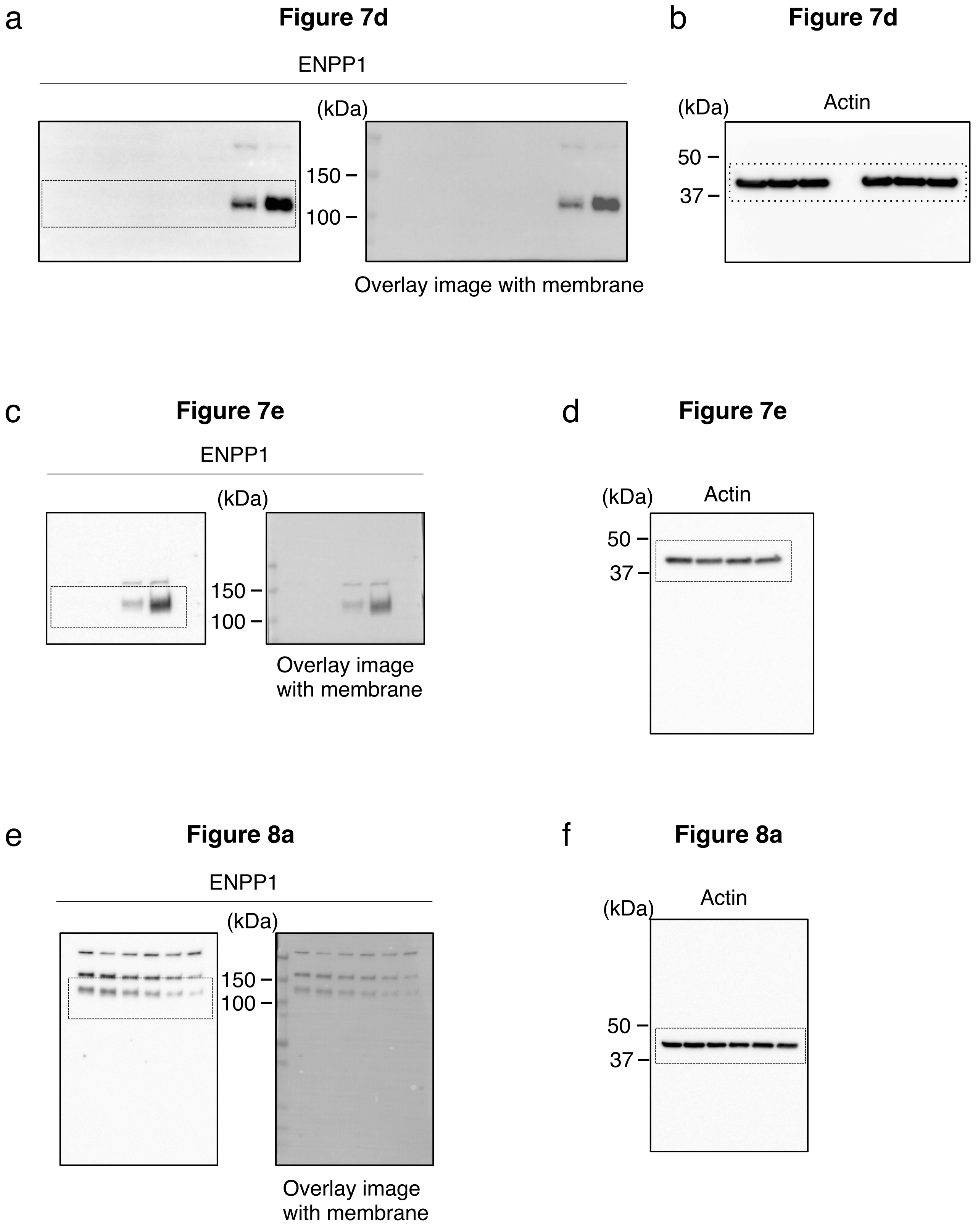

Supplementary Fig. 15. Original data of western blot analysis.

Supplementary Table 1

Patient ID	Recurrence	Subtype (Luminal)
1	non recurrence	A
2	non recurrence	A
3	recurrence	B
4	non recurrence	A
5	recurrence	B
6	non recurrence	A
7	recurrence	B
8	recurrence	A
9	recurrence	B
10	recurrence	A
11	recurrence	B
12	recurrence	B
13	non recurrence	B
14	recurrence	A
15	non recurrence	B
16	non recurrence	A
17	non recurrence	B
18	non recurrence	A
19	recurrence	A
20	non recurrence	A
21	recurrence	B
22	recurrence	B
23	non recurrence	A
24	non recurrence	A
25	non recurrence	A
26	non recurrence	B

Clinical information of breast cancer patients in NCC

Supplementary Table 1. Clinical parameters of the breast cancer patients included in the study.

Supplementary Table 2

			up-regulated genes : Fold change (> 2-fold, $p < 0.05$)						down-regulated genes : Fold change (< 0.7-fold, $p < 0.05$)		
			MCF7-luc anti-NC			MCF7-luc anti-miR-27b			MCF7-luc miR-27b o.e		
rank	GeneName	SystematicName	Normalized	Raw	FoldChange	Normalized	Raw	FoldChange	Normalized	Raw	FoldChange
1	UGT2B15	NM_001076	0.171823992	70.500435	1	6.803261294	2553.0068	39.59436176	0.086143634	32.29355	0.501348114
2	UGT2B11	NM_001073	0.171146738	70.22255	1	3.593965351	1348.6808	20.99932137	0.108496138	40.67306	0.63393635
3	ENPP1	NM_006208	0.085093503	34.914383	1	1.37263545	515.0987	16.13090773	0.053297941	19.980349	0.626345591
4	PP14571	NR_024014	0.056929084	23.358343	1	0.881845047	330.92322	15.49023773	0.030569865	11.46004	0.536981503
5	TNFRSF11B	NM_002546	0.11758256	48.244843	1	1.778106577	667.2568	15.12219649	0.056300362	21.10589	0.47881558
6	TNFRSF11B	NM_002546	0.122226414	50.15024	1	1.845322535	692.48016	15.09757568	0.07817437	29.306038	0.639586548
7	FAM189A2	NM_004816	0.090335083	37.065037	1	1.292743186	485.11792	14.31053298	0.055410327	20.772242	0.613386577
8	TNFRSF11B	NM_002546	0.137708164	56.502502	1	1.922388869	721.4	13.95987579	0.085222247	31.948132	0.618861252
9	TNFRSF11B	NM_002546	0.142341732	58.40369	1	1.890044628	709.26245	13.27821857	0.086547413	32.444923	0.608025569
10	TNFRSF11B	NM_002546	0.142962849	58.65853	1	1.802740326	676.50085	12.60985167	0.061049953	22.886427	0.427033691
11	TNFRSF11B	NM_002546	0.160841578	65.99428	1	1.906004466	715.25183	11.85019751	0.078190433	29.312054	0.48613321
12	LHFP	NM_005780	0.130971353	53.738346	1	1.359304761	510.09604	10.37864178	0.067282037	25.222712	0.513715675
13	ENTPD8	NM_001033113	0.193029025	79.20099	1	1.871061203	702.1387	9.693159897	0.128777951	48.276314	0.667142937
14	DHRS2	NM_182908	3.394289693	1392.6978	1	32.83051327	12320.057	9.672277926	1.722935269	645.8944	0.507598179
15	TIMP3	NM_000362	0.102712787	42.14368	1	0.847886664	318.18008	8.254928046	0.036234304	13.583528	0.352773061
16	KRTAP3-1	NM_031958	0.140965146	57.83886	1	1.047291309	393.00903	7.429434435	0.079545399	29.820005	0.564291256
17	KRTAP3-1	NM_031958	0.149649794	61.40222	1	0.964022382	361.7614	6.441855717	0.095142728	35.66714	0.635769186
18	PTGER4	NM_000958	0.399231096	163.80688	1	2.393677017	898.2565	5.995717876	0.262617325	98.450096	0.657807791
19	TFF3	NM_003226	0.920477767	377.67783	1	5.279932918	1981.36	5.736078707	0.517156093	193.87164	0.561834421
20	PTPN21	NM_007039	0.431412176	177.011	1	2.391293147	897.36273	5.542943108	0.261688041	98.10169	0.606584736
21	FAM129A	NM_052966	0.170983901	70.15574	1	0.922551024	346.19864	5.395543195	0.11053901	41.438915	0.646487822
22	TFF3	NM_003226	14.9351125	6127.968	1	79.50953624	29836.94	5.323665036	5.919430981	2219.0793	0.396343247
23	GPC6	NM_005708	0.105244358	43.182404	1	0.515525659	193.45738	4.8983686	0.054362466	20.379412	0.516535675
24	AK094885	AK094885	0.087153765	35.75972	1	0.408925178	153.45425	4.691996704	0.05102245	19.127312	0.58543025
25	TFF3	NM_003226	76.46270321	31373.123	1	356.9464887	133948.48	4.668243127	30.72380515	11517.751	0.401814268
26	SERPINA3	NM_001085	0.131265619	53.85908	1	0.597163267	224.09294	4.549274	0.045063847	16.893549	0.343302745
27	EPB41L2	NM_001431	0.064537267	26.48004	1	0.291786755	109.49659	4.521213356	0.032943051	12.349697	0.510450051
28	PRSS23	NM_007173	0.750971665	308.1283	1	3.355039612	1259.0206	4.467598136	0.473390021	177.46458	0.630370017
29	EPHA4	NM_004438	1.2391712	508.43942	1	5.3652396	2013.3721	4.329700043	0.803630336	301.26514	0.648522444
30	EPHA4	NM_004438	1.198946597	491.93494	1	5.113476961	1918.8965	4.264974749	0.802452733	300.82376	0.669298145
31	EPHA4	NM_004438	1.157666948	474.9978	1	4.919559017	1846.1251	4.249546061	0.74135299	277.91864	0.640385382
32	KRT32	NM_002278	0.232965173	95.58703	1	0.983189459	368.95398	4.220328072	0.155631554	58.343204	0.668046439
33	EPHA4	NM_004438	1.238061971	507.98453	1	5.220335758	1958.9961	4.216538332	0.788284657	295.51245	0.636708562
34	NRCAM	NM_001037132	0.756246193	310.29248	1	3.185705711	1195.4761	4.212524626	0.230725122	86.4943	0.305092606
35	EPHA4	NM_004438	1.241095816	509.2292	1	5.18871214	1947.1288	4.18075065	0.804057555	301.4253	0.647860983
36	ADM	NM_001124	1.63416516	670.50793	1	6.509085207	2442.6145	3.983125675	0.629032542	235.81189	0.384925929
37	ADM	NM_001124	1.816843559	745.4621	1	7.115827808	2670.303	3.916588071	0.677379096	253.93611	0.372832924
38	NEFH	NM_021076	0.125081298	51.321617	1	0.479550083	179.95706	3.833907155	0.080573855	30.205551	0.644171885
39	ADM	NM_001124	1.778208014	729.60944	1	6.701551789	2514.8406	3.768710824	0.680892619	255.25328	0.382909431
40	ADM	NM_001124	1.798189992	737.8079	1	6.753095688	2534.182	3.755496204	0.681275297	255.39674	0.37886725
41	ADM	NM_001124	1.80274866	739.6788	1	6.65968343	2499.1287	3.694182986	0.6898642	258.6165	0.382673534
42	ADM	NM_001124	1.688242092	692.6958	1	6.151666105	2308.4893	3.643829361	0.650337238	243.79866	0.385215628
43	ADM	NM_001124	1.825600384	749.05524	1	6.643480669	2493.0486	3.63906621	0.659841078	247.3614	0.36143785
44	LMO2	NM_005574	0.453496839	186.07256	1	1.626638922	610.4163	3.586880397	0.278231275	104.30343	0.613524176
45	RGAG4	NM_001024455	0.402949542	165.33266	1	1.418184762	532.1914	3.519509548	0.240257038	90.06762	0.596245964
46	LMO2	NM_005574	0.457166248	187.57812	1	1.583777204	594.3319	3.464335372	0.276912503	103.80904	0.605715107
47	FGF13	NM_004114	0.082078405	33.67727	1	0.27955683	104.907196	3.405973004	0.050146576	18.798962	0.610959435
48	NRCAM	NM_001193582	0.254636282	104.478806	1	0.862461762	323.64948	3.387034073	0.09525682	35.70991	0.374089739
49	EDN1	NM_001955	4.307102927	1767.2314	1	14.48592394	5436.026	3.363263935	2.864144142	1073.7109	0.664981588
50	LMO2	NM_005574	0.458303021	188.04456	1	1.540295945	578.0153	3.360867974	0.26935443	100.97565	0.587721263

Supplementary Table 2. Identification of miR-27b targets.

Candidate miR-27b target genes that were up-regulated in MCF7-luc anti-miR-27b cells and down-regulated in MCF7-luc miR-27b o.e. cells compared with MCF7-luc cells. The total RNA was labelled with Cy3 using the Low Input Quick Amp Labeling Kit (Agilent) and hybridized to a SurePrint G3 Human GE 8×60 K array (Agilent), according to the manufacturer's protocol. Data analysis was performed using GeneSpring software.

Supplementary Table 3

Fold change (> 2fold, *p* <0.05) mRNA up-regulated genes mRNA up-regulated genes

		anti-miRNC			anti-miR27b			ENPP1 o.e.		
GeneName	SystematicName	Normalized	Raw	FoldChange	Normalized	Raw	FoldChange	Normalized	Raw	FoldChange
AKR1C3	NM_003739	0.149627141	61.392925	1	2.054944172	771.143	13.73376616	0.360931284	136.1707	2.412204634
DSCR8	NR_026838	0.236026279	96.84305	1	3.21561949	1206.701	13.62398928	1.125075095	424.46387	4.766736576
BCAS1	NM_003657	1.747600399	717.0507	1	20.98811216	7876.044	12.00967463	4.37911337	1652.134	2.505786434
MAPK4	NM_002747	0.092393246	37.909508	1	1.015577433	381.10788	10.99190125	0.354580499	133.7747	3.837731821
BCAS1	NM_003657	0.519004924	212.95088	1	5.496059413	2062.4648	10.58960939	1.146748563	432.64075	2.209513843
KCNJ8	NM_004982	0.196370484	80.57203	1	2.033291134	763.0178	10.35436231	0.471238103	177.78683	2.399739991
HMCN1	NM_031935	0.235001798	96.42267	1	2.187489361	820.8826	9.308394145	0.470137446	177.37154	2.000569571
ZMAT4	NM_024645	0.081260232	33.341564	1	0.726459251	272.61282	8.939911069	0.164984626	62.24473	2.030324321
CSTA	NM_005213	0.046082455	18.907911	1	0.405903982	152.32053	8.808210823	0.124578758	47.000572	2.703388023
XLOC_010305	TCONS_00021722	0.368664856	151.26544	1	3.102543633	1164.268	8.415620802	1.241005578	468.20157	3.36621611
TIMP3	NM_000362	0.102712787	42.14368	1	0.847886664	318.18008	8.254928046	0.269281925	101.59359	2.621698168
ABCG2	NM_004827	0.065300018	26.792995	1	0.499703144	187.51979	7.652419695	0.171864205	64.84023	2.631916648
LOC100506418	XR_109447	0.073837955	30.296167	1	0.55688236	208.97702	7.541952687	0.161172421	60.80648	2.182785553
XLOC_010305	TCONS_00021720	0.055807182	22.898027	1	0.385860856	144.79906	6.914179222	0.173984103	65.64	3.117593438
SGCG	NM_000231	0.252216489	103.485954	1	1.576340281	591.54126	6.249949358	0.595908652	224.82198	2.362687133
DLG2	NM_001364	0.05555643	22.795141	1	0.346236662	129.92961	6.232161846	0.218778064	82.53973	3.937943194
TGFB2	NM_003238	0.039492117	16.203861	1	0.228624782	85.79425	5.789124567	0.180370174	68.04931	4.567245057
ERBB4	NM_005235	0.872086509	357.82242	1	5.030127609	1887.6178	5.767922739	2.906011274	1096.368	3.33225115
GRHL3	NM_198173	0.168569917	69.16526	1	0.891002769	334.35983	5.285657045	0.355367216	134.0715	2.10812951
SLC24A3	NM_020689	1.344397933	551.6144	1	6.948427894	2607.4846	5.168430955	2.793385527	1053.8768	2.077796654
ENST000005568	ENST00000556886	0.029173864	11.970214	1	0.147155595	55.22195	5.044089939	0.0981134	37.015835	3.363058093
XLOC_013670	THC2687906	0.03403558	13.965004	1	0.169344895	63.54875	4.975525477	0.098089716	37.006878	2.881975758
XLOC_l2_00654	TCONS_l2_00012221	0.034540929	14.172354	1	0.166251516	62.387943	4.813174404	0.081018108	30.566185	2.345568282
ENST000004144	ENST00000414452	0.102455639	42.038174	1	0.480569702	180.33975	4.690514891	0.226383701	85.409134	2.209577754
HS6ST3	NM_153456	0.321930194	132.08989	1	1.285601012	482.4377	3.993415453	0.774582064	292.23123	2.406055965
ENST000003988	ENST00000398832	0.02911165	11.944689	1	0.116235028	43.618633	3.992732446	0.058262443	21.981016	2.001344603
PCDH9	BC150296	0.257209654	105.53471	1	0.978963013	367.36795	3.806089688	0.575290495	217.04327	2.236659805
DLG2	NM_001364	0.025783984	10.579326	1	0.098121391	36.82127	3.805517092	0.101180053	38.172802	3.924143501
MESP1	NM_018670	0.933610835	383.0661	1	3.549145451	1331.8608	3.801525557	2.751876642	1038.2166	2.947562879
ERP27	NM_152321	0.092792776	38.07345	1	0.350922048	131.68785	3.781781995	0.233651467	88.151085	2.517992001
LIPH	NM_139248	0.224710431	92.20006	1	0.848718971	318.49237	3.776945143	0.461557758	174.13463	2.054011274
RFPL1	NM_021026	0.075166144	30.841125	1	0.282834693	106.13722	3.762793716	0.171274182	64.61763	2.278608076
RFPL2	NM_006605	0.164535523	67.50995	1	0.532336923	199.76598	3.235392046	0.337522738	127.33923	2.051366971
PAPSS2	NM_001015880	0.678265088	278.29626	1	2.122980029	796.6747	3.130015193	1.457128894	549.7397	2.148317701
PSCA	NM_005672	0.261382725	107.24689	1	0.812687326	304.97098	3.109185299	0.777238968	293.2336	2.973566706
AKR1C1	NM_001353	0.161133403	66.11403	1	0.481239577	180.5911	2.98659104	0.406073025	153.20158	2.520104572
XLOC_l2_00783	ENST00000426962	0.057212492	23.474638	1	0.170344751	63.923977	2.977404842	0.126183484	47.606	2.205523297
XLOC_000004	ENST00000522481	0.18108286	74.2994	1	0.531215125	199.34505	2.933547251	0.381613893	143.97371	2.107399307
CAPN5	NM_004055	0.530297995	217.58456	1	1.540716262	578.1728	2.905378251	1.114713685	420.5546	2.102051477
XLOC_005749	ENST00000425089	0.163693847	67.16458	1	0.453156442	170.05254	2.768316894	0.347468235	131.09143	2.122671323
XLOC_004745	ENST00000511182	0.024862273	10.201143	1	0.068752501	25.80023	2.765334496	0.084838851	32.00766	3.412353005
XLOC_l2_00106	ENST00000430316	0.028600734	11.735057	1	0.078447022	29.438213	2.742832458	0.089443745	33.74498	3.127323362
C18orf1	NM_181482	0.166409419	68.27879	1	0.453207236	170.07166	2.723447016	0.343170251	129.4699	2.062204492
PMEL	NM_006928	0.598856771	245.7146	1	1.567657972	588.2832	2.6177511	1.533701019	578.62854	2.561048137
AKR1C1	NM_001353	0.366608032	150.42152	1	0.946718953	355.268	2.582373734	0.926557504	349.5678	2.527379169
LOC100505909	XR_109346	0.197126631	80.882256	1	0.503216743	188.83829	2.552758801	0.424458909	160.13818	2.153229655
AKR1CL1	NR_027916	0.138118675	56.67094	1	0.341791542	128.26155	2.474622224	0.285923975	107.872215	2.070132627
PGM5	NM_021965	0.281038269	115.311714	1	0.684582491	256.89813	2.435904883	1.232650071	465.04916	4.386057727
LOC100505909	XR_109346	0.289384649	118.736305	1	0.681544943	255.75812	2.35515237	0.615429784	232.18683	2.126684277
LOC572558	NR_015423	0.067034742	27.504763	1	0.154819242	58.09786	2.309537386	1.059538675	399.73828	15.80581421
LOC100505909	XR_109346	0.248859437	102.10854	1	0.570797808	214.19899	2.293655469	0.516181092	194.74275	2.074187334
LOC729468	BU567215	0.124696856	51.163868	1	0.279183793	104.767204	2.238900012	0.593488084	223.90874	4.759447044
LOC100506299	ENST00000455965	0.174487368	71.59323	1	0.382070109	143.37656	2.189672032	0.38392042	144.84396	2.200276297
PHLDA1	NM_007350	0.072975095	29.942125	1	0.159190153	59.73808	2.18143124	0.18756147	70.76246	2.570212066
NR4A2	NM_006186	0.138068164	56.65021	1	0.300022936	112.58731	2.173005913	0.352754037	133.08563	2.554926698
MICB	NM_005931	1.33501708	547.766	1	2.726034461	1022.9784	2.041947254	3.757195015	1417.4994	2.814342281
NR4A2	NM_006186	0.166560129	68.34064	1	0.338890171	127.17276	2.034641617	0.345017704	130.16687	2.071430333
UGT1A6	NM_001072	0.090661629	37.199017	1	0.183931413	69.02253	2.028768007	1.796887392	677.92267	19.81971209

Supplementary Table 3. Identification of ENPP1-regulated genes.
Genes that were commonly up-regulated in MCF7-luc anti-miR-27b and MCF7-ENPP1-MF cells.

Supplementary Table 4

Primer name	Nucleotide sequence
For qPCR	
GAPDH Fw	TGAAGGTCGGAGTCAACGATTTGGT
GAPDH Rv	GAAGATGGTGATGGGATTTC
ENPP1 Fw	GAACTTGCACAGATGCCTGA
ENPP1 Rv	GGGACATCAGAGGGTCTCAA
ABCG2 Fw	AGCTGCAAGGAAAGATCCAA
ABCG2 Rv	TCCAGACACACCACGGATAA
For cloning	
Cloning for 3’UTR of ENPP1 Fw	AAAGCGGCCGCAGAGTTAGAACGGAGCCCTCGG
Cloning for 3’UTR of ENPP1 Fw	TTTCTCGAGGCCCTTAGGCCGTTGAAGAATGGT
ENPP1 3’UTR mutation Fw	AAGTAGTTACTCCCATTGTCTGGATACCAGATATTTGAATCTTTCTTAC
ENPP1 3’UTR mutation Rv	ACAATGGGAGTAACTACTTCTCCTTTAAGAGAAGCAGAAAGGCAG

Supplementary Table 4a: Primer sequences used for this study.

1. anti-miR-27b (miR-ZIP-27b)<br>5’-GGATCCGTTCACAGCGGCTAAGTCCTAC-CTTCCTGTCAG-GCAGAACTTAGCCACTGTGAATTTTTGAATTC-3’
2. anti-miR-23b (miR-ZIP-23b)<br>5’-GGATCCGATCACATCGCCAGGGACTAACCTTCCTGTCAG-GGTAATCCCTGGCAATGTGATTTTTTGAATTC-3’
3. Scramble Hairpin Control - Construct (for miRZip)<br>5’-gGATCCCTAAGGTTAAGTCGCCCTCGCTCTAGCGAGGGCGACTTAACCTTAGGTTTTTGAATTc-3’
4. miR-27b o.e.<br>The sequence of hsa-mir-27b precursor was inserted.<br>5’-AGAACAGGTGCATCTCGTAGCTCTTCTTTGGAAACAAAAGAAGCCACCAGCTGAGGAAGATGCTCACCGG<br>TCACCGTCCCTTTATTTATGCCCAGCGATGACCTCTCTAACAAGGTGCAGAGCTTAGCTGATTGGTGAACA<br>→ miR-27b-3p sequence<br>GTGATTGGTTTCCGCTTTGTTCACAGTGGCTAAGTTCTGCACCTGAAGAGAAGGTGAGATGGGGACAGTTA<br>AGTTGGAGCCGCTGGGGCAGAGGCCGTTGCTGACGGGCCGGCCGCTGCTGCACAGTCAGCTTGGGTGC<br>GGAGCGCGATCCTGGAG-3’

Supplementary Table 4b: Details of constructs for miR-27b knockdown and overexpression.

miRCURY LNA™ microRNA Power Inhibitor (EXIQON)
1. LNA miR-27b (hsa-miR-27b-3p): AGAACTTAGCCACTGTGA
2. Control LNA: TAACACGTCTATACGCCCA

Supplementary Table 4c: The sequences of LNA for miR-27b knockdown.
